# Additively manufactured porous scaffolds by design for treatment of bone defects

**DOI:** 10.3389/fbioe.2023.1252636

**Published:** 2024-01-19

**Authors:** Shirin Toosi, Mohammad Javad Javid-Naderi, Ali Tamayol, Mohammad Hossein Ebrahimzadeh, Sima Yaghoubian, Seyed Ali Mousavi Shaegh

**Affiliations:** ^1^ Stem Cell and Regenerative Medicine Center, Mashhad University of Medical Science, Mashhad, Iran; ^2^ Department of Medical Biotechnology and Nanotechnology, Faculty of Medicine, Mashhad University of Medical Science, Mashhad, Iran; ^3^ Department of Biomedical Engineering, University of Connecticut Health Center, Farmington, CT, United States; ^4^ Orthopedic Research Center, Ghaem Hospital, Mashhad University of Medical Sciences, Mashhad, Iran; ^5^ Laboratory for Microfluidics and Medical Microsystems, BuAli Research Institute, Mashhad University of Medical Science, Mashhad, Iran; ^6^ Clinical Research Unit, Ghaem Hospital, Mashhad University of Medical Science, Mashhad, Iran

**Keywords:** additive manufacturing, 3D printing, triply periodic minimal surface, scaffold, bone defect, regeneration

## Abstract

There has been increasing attention to produce porous scaffolds that mimic human bone properties for enhancement of tissue ingrowth, regeneration, and integration. Additive manufacturing (AM) technologies, i.e., three dimensional (3D) printing, have played a substantial role in engineering porous scaffolds for clinical applications owing to their high level of design and fabrication flexibility. To this end, this review article attempts to provide a detailed overview on the main design considerations of porous scaffolds such as permeability, adhesion, vascularisation, and interfacial features and their interplay to affect bone regeneration and osseointegration. Physiology of bone regeneration was initially explained that was followed by analysing the impacts of porosity, pore size, permeability and surface chemistry of porous scaffolds on bone regeneration in defects. Importantly, major 3D printing methods employed for fabrication of porous bone substitutes were also discussed. Advancements of MA technologies have allowed for the production of bone scaffolds with complex geometries in polymers, composites and metals with well-tailored architectural, mechanical, and mass transport features. In this way, a particular attention was devoted to reviewing 3D printed scaffolds with triply periodic minimal surface (TPMS) geometries that mimic the hierarchical structure of human bones. In overall, this review enlighten a design pathway to produce patient-specific 3D-printed bone substitutions with high regeneration and osseointegration capacity for repairing large bone defects.

## 1 Introduction

For the restoration of complex, critically sized difficult-to-heal, or non-healing bone damages and defects, there is no ideal solution. The current clinical approach is to employ bone autografts, allografts, or bone fillers at a defect site; however, the efficacy of such methods for bone regeneration depends on the defect size, its anatomical position, bone nature, and patient’s underlying conditions (Buttery and Bishop, 2005). These strategies are typically inadequate to regenerate all bone defects, and carry the risk of adverse immune response and diseases transmission. Tissue engineering (TE) methods have been developed to overcome these limitations of bone reconstruction through development of implantable porous scaffolds (Puppi et al., 2010; Henkel et al., 2013; Roseti et al., 2017). Scaffolds should be able to recreate the structure of the lost tissue, facilitate the regeneration, offer mechanical properties supporting tissue function, and eventually integrate with the native tissue (Hollister and Murphy, 2011; Ghelich et al., 2022). Bone tissue engineering scaffolds have been fabricated using different methods including gas foaming (Liu and Ma, 2004; Salerno et al., 2012; Bak et al., 2014; Poursamar et al., 2016), laser sintering (Williams et al., 2005; Duan et al., 2010; Maskery et al., 2018), electrospinning (Liao et al., 2008; Jang et al., 2009; Prabhakaran et al., 2009; Di Martino et al., 2011; Yu et al., 2016; Lin et al., 2020), and recently, additive manufacturing (AM) (Valainis et al., 2012; Mota et al., 2015; Bobbert et al., 2017; Bobbert et al., 2017; Düregger et al., 2018; Zadpoor, 2019a; Zadpoor, 2019b; Soro et al., 2019; Yuan et al., 2019). Among them, AM tools including 3D printing have been explored to fabricate scaffolds with controlled architectural, topological, biological, mechanical, and mass transport features (Bobbert et al., 2017; Faramarzi et al., 2018; Ostrovidov et al., 2019; Maydanshahi et al., 2021) to mimic the features of native bones.

Due to the staggering number of patients suffering from bone fracture and loss, the materials and fabrication processes used for bone scaffolds have been critically reviewed elsewhere and will not be discussed in detail here. One area that has not been properly reviewed is the importance of the pore geometrical features on the bone tissue regeneration. In the following sections, initially, a brief overview of the physiology of bone regeneration is provided to provide a better understanding of scaffold design considerations in practice. Then, effects of design parameters on various features of scaffold with a particular emphasis on structures having triply periodic minimal surface (TPMS) geometries are highlighted. TPMS structures provide a higher surface-area-to-volume ratio in comparison with conventional lattice structures that promote cell adhesion, cell migration and cell proliferation. In overall, the challenges limiting the fabrication of scaffolds by design and the opportunities for overcoming these barriers are discussed.

## 2 Bone regeneration physiology and pathophysiology of large bone defects

Bone is considered as a rigid organ in body which support and guard some other organs and facilitate mobility of live body (Martin et al., 2008; Wang and Yeung, 2017). Bone is a porous composite material, which could be divided into categories of (i) compact and (ii) cancellous bones. Compact section is a hard outer-shell of bone (compact bone), which possesses lower porosity, while cancellous section has a highly porous structure inside a bone (cancellous bone), which is less dense than the outer surface. Porosity of compact bone is within the range of 5%–10% with apparent density of 1.5–1.8 g/cm^3^; that is the reason why it is called “compact” bone. Cancellous bones have 30%–95% porosity with pores sizes range from 200 μm to 1,000 µm. A desirable porous bone substitute is the one that mimics human bone properties with hierarchical architecture which enable tissue ingrowth and movement of bodily fluids through itself that are required for cell proliferation (Bobbert et al., 2017). Bone is mostly made of hard apatite minerals along with soft collagen protein networks (Currey, 1969). Such composite construction generates the stiffness and the suitable function of bone tissue. For instance, ear bone content of over 80% mineral allows its vibration to transmit sound, however, it is unable to resorb energy (Wang and Yeung, 2017). On the other hand, deer antlers consist of less dense mineral content for absorbing high energy levels (Wang and Yeung, 2017).

**FIGURE 1:**
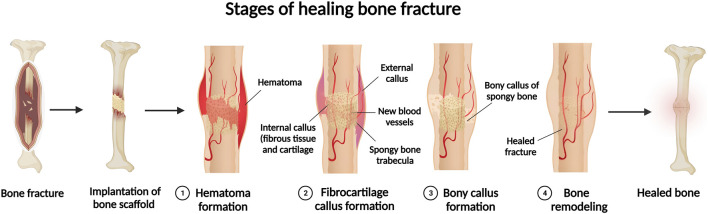
Schematics for bone healing process with implantation of scaffold in bone fracture.

Bone formation is continued dynamically through two different processes, known as modelling and remodelling (Wang and Yeung, 2017) that also contribute to bone fracture recovery. Bone modelling progression starts with formation of a new bone with no prior bone resorption, however, during the course of bone remodelling, bone formation occurs in following with bone resorption (Wang and Yeung, 2017). Bone modelling starts at the early ages, changes the shape and size of body bones as it grows to. Bone modelling stops once body reaches its adult age (Kimmel, 1993). In contrast, remodelling is a lifelong procedure, which starts at early life and maintains bone health for proper functionality by constantly substituting impaired bone with new bone (Kimmel, 1993). In contrary to other tissues, bone healing enables body to repair a damaged bone and fully restore it to its previous composition, construction, and functionality (Einhorn and Gerstenfeld, 2015). Bone repair can be defined into direct, i.e., primary bone healing, as well as indirect or secondary bone healing procedures. Direct bone healing mainly starts when small and narrow gaps, usually less than 0.1 mm fractures, happen and the fraction site is rigidly stabilized. As direct bone healing progresses, bone gap is filled continuously through ossification and following Haversian remodelling (DeLacure, 1994). Indirect bone healing happens once the fracture edges are smaller than twice the injured bone diameter. It includes several actions, such as the formation of blood clot, inflammatory response, and formation of fibro-cartilage callus at the site of injury; as well as intramembranous and endochondral ossification, and bone remodelling. The bone fracture repair mechanism initiates with anabolism, increasing bone, differentiation of recruiting stem cells, and retardation with chondrocyte apoptosis (Lee et al., 1998; Einhorn and Gerstenfeld, 2015). Some event such as high-energy trauma, disease, revision and secondary surgeries, developmental deformities, and tumour resections can deteriorates bone healing and create large segmental bone defects (Gugala and Gogolewski, 2002; Wildemann et al., 2007; Reichert et al., 2009). These large bone losses can affect blood circulation and tissue differentiation that finally can lead to bone fracture, that may result in non-union without interventions (Claes et al., 2003). In addition, defect size is not the only parameter that determines a critical bone defect (Lindsey et al., 2006), but defect length is also an important factor that should be considered for bone healing (Khan et al., 2005; Lindsey et al., 2006). Regarding the impressive improvement in the field of bone healing, still non-properly healed fractures or bone defects can extremely affect the quality of patients’ lives because of treatment costs and prolong period of healing (Figure 1).

## 3 Role of porous scaffolds as bone substitute in bone tissue engineering

**FIGURE 2:**
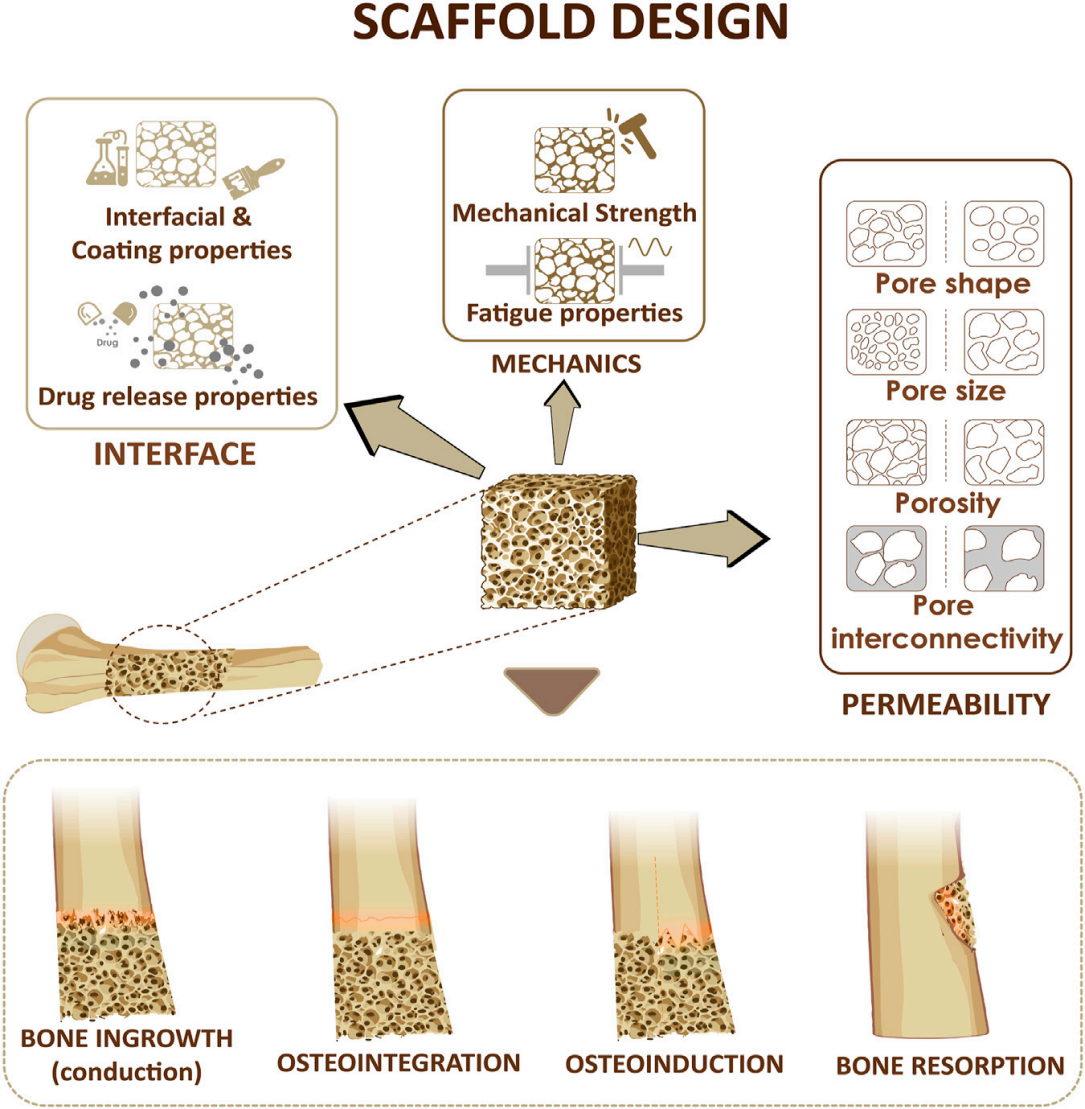
Design considerations for a bone scaffold. Three parameters including scaffold permeability, mechanical strength of the scaffold and the interfacial adhesion at the interface will affect bone ingrowth, osteoinduction, osteointegration, bone resorption.

**FIGURE 3:**
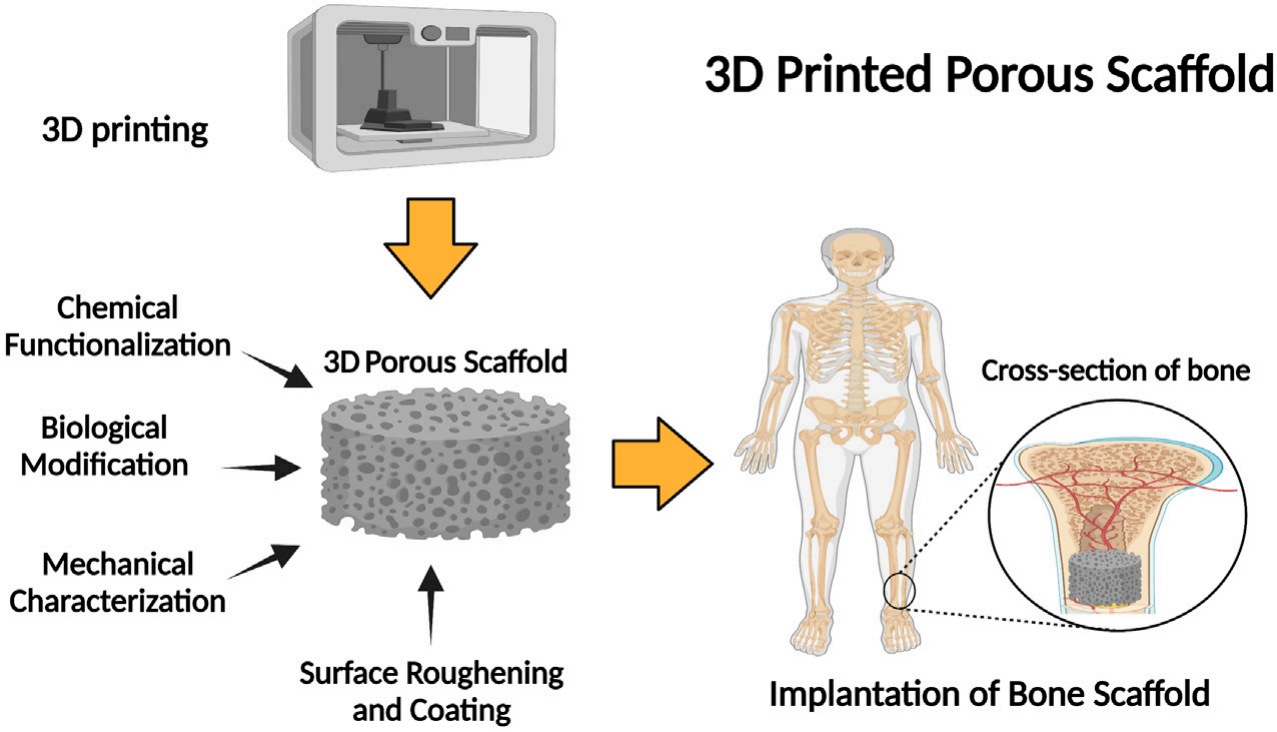
Schematic illustration of the fabrication of 3D porous scaffolds: properties and application.

In addition to the properties of bulk materials used for scaffolds preparation, the architectural features of scaffolds affect their function and characteristics. For example, porosity, pore size distribution, and their interconnectivity affect the transport properties and the mechanical properties (Figures 2, 3). In the following section, we will discuses the effects of porosity on various characteristics of scaffolds.

### 3.1 Effect of porosity, pore size and porous scaffold on bone regeneration

Size and geometry of the scaffold’s pores, as well as interconnectivity of porous structure highly affect cellular penetration and distribution, their proliferation and differentiation, and formation of blood vessels.

Osteoblasts size ranges within 10–50 μm (Sugawara et al., 2005), however, larger pores (100–200 μm) are more suitable for their function to regenerate mineralised bone post implantation (Toosi et al., 2016; Abbasi et al., 2020). In this way, macrophages are allowed to infiltrate and eliminate bacteria. In addition, infiltration of other cells involved in colonisation, migration and vascularisation is supported (Iviglia et al., 2019).

However, smaller pore size (<100 μm) may induce the creation of non-mineralised osteoid or fibrous tissue (Liu et al., 2018; Iviglia et al., 2019). Previous studies reported that significant bone formation was observed in scaffolds with 800-μm pore sizes. Fibroblasts preferred to fill smaller pores while larger pores were filled bone cells revealing a 800 μm-scaffold could be more suitable for homing and ingrowth of bone cells (Roosa et al., 2010). In a study by Karageorgiou and Kaplan (2005), scaffolds with pore sizes greater than 300 μm were found to be more appropriate to repair large bone defects as the formation of new bone and capillaries were enhanced (Hollister and Murphy, 2011). Excellent osteoinductions were reported for scaffolds having pore sizes ranges from 500 μm to 1,200 μm (Hutmacher, 2000; Van Bael et al., 2012).

Similar observation has been made in the case of hydrogel-based scaffolds. Further, pore size significantly affects individual cell’s response including its attachment, growth as well as proliferation (Al-Munajjed et al., 2008). Highly porous scaffolds are easier for cells to penetrate since material degradation through expression of matrix metalloproteinase (MMPs) is not needed to create space for cell migration. This can potentially reduce the level of inflammation as the over expression of MMPs can induce inflammatory responses (Rosso et al., 2005; Anderson et al., 2011). However, for scaffolds that are expected to support bone regeneration throughout their volume, pore size distribution and their interconnectivity are critical features. In another study, it was shown that bone cells grow faster and differentiate in scaffolds with pores within the range of 100 μm–325 µm (Abbasi et al., 2020). Another important study was performed on scaffolds with small (90–120 µm) and large (350 µm) pore diameters as implanted in rats. Scaffolds with small pores showed chondrogenesis before osteogenesis, while in those with larger pores, direct bone formation was observed due to enhanced vascularization through the pores. The vascularization observed in the larger pores facilitated mass transport through the pores for sufficient oxygen and nutrient delivery as required for direct osteogenesis. Cheng et al. (2016) employed magnesium scaffolds in pore sizes of 250 μm and 400 μm. They observed that formation of mature bone was more in the larger pores owing to improved vascularisation. In this way, sufficient level of oxygen and nutrients could be delivered to maintain osteoblastic activity that result in upregulation of osteopontin (OPN) and collagen type I with direct impact on augmentation of bone mass (Cheng et al., 2016).

In another study, Lim et al. (2010) testified that 200 μm–350 μm was optimum size for osteoblast proliferation whereas cell attachment was not affected at pores with larger sizes (500 μm). Cell aggregation and proliferation could be more controlled in smaller pores (Chen et al., 2018), however, such scaffolds may stimulate endothelial cell proliferation due to exogenous hypoxic condition (Bianco et al., 2017). In addition, proinflammatory higher levels of cytokines including tumour necrosis factor α and interleukin 6, 10, 12, and 13 could be produced in pores with larger size that can activate bone regeneration responses (Mukherjee et al., 2019).

Micropores, however in contrast to macropores, promote protein adhesion and cell attachment over the scaffolds *in vitro* (Wang et al., 2016; Diaz-Rodriguez et al., 2018; Sokolova and Epple, 2021). O’Brien et al. (2005) found that pores with size of 95 μm could provide the best environment for initial cell adhesion at *in vitro* conditions (Mukherjee et al., 2019). In another study it was reported that scaffolds with pore size of 100 μm–325 μm was optimal for bone engineering *in vitro* (Mukherjee et al., 2019). Some previous studies claimed that although pores larger than 50 μm (i.e., >50 μm, macropores) provide favourable effects to enhance osteogenic quality; cell infiltration is limited in small pore size at *in vitro* conditions.

Owing to these facts, it could be concluded that scaffolds designed to have a gradient in pore size and porosity might provide an optimal solution for bone regeneration. Gradient PCL scaffolds could enhance the osteogenic differentiation of human mesenchymal stem cells (MSCs) at *in vitro* through increased level of calcium content and ALP activity as a consequence of improved supply of oxygen and nutrients in larger pores (Di Luca et al., 2016). Effect of gradient porosity on cell-seeding was evaluated by Sobral et al. (2011) on 3D poly (ε-caprolactone) scaffolds with pore size of 100–700–100 μm and 700–100–700 μm. In static conditions, the gradient porosity showed higher seeding efficiency, as increased from 35% for uniform porosity to about 70% in the gradient pore sizes (Sobral et al., 2011). In addition, for degradable scaffolds, pore size and porosity regulate the degradation rate. As for PLA scaffolds, Xu et al. (2014) reported that square shape pores had higher degradability and scaffold weight loss.

Porosity is a morphological property of a porous structure and is independent of the structure material that highly affects the biological response of scaffolds (Karageorgiou and Kaplan, 2005; Lv et al., 2021). Such interconnectivity is one of the most essential requirements for tissue ingrowth (Lee et al., 2019; Ferrández-Montero et al., 2020; Dong et al., 2021; Jamee et al., 2021; Jiang et al., 2021; Kumar et al., 2021).

Porosity (P), defined as void space percentage of a solid structure, is determined by Eq. 1 as shown in below (Léon and León, 1998; Yuan et al., 2019):

$$P=\left(1-\frac{P\;structre}{P\;material}\right)\times 100\%$$ (1)

where *P*_material_ shows the density of the bulk material and *P*_structure_ is the density of the porous structure (Karageorgiou and Kaplan, 2005).

### 3.2 Effect of porosity and pore size on permeability of porous structures

The microstructure of TE scaffolds is generally characterized by porosity, pore size, interconnectivity and tortuosity. However, these parameters are not sufficient to predict the success of a porous scaffold. On the other hand, permeability is an important parameter in the assessment of biological performance, including mass transport parameter and can be considered as an independent design parameter (Kemppainen and Hollister, 2010; Pennella et al., 2013; Lipowiecki et al., 2014; Ali and Sen, 2017; Montazerian et al., 2017; Rahbari et al., 2017; Daish et al., 2019; Lv et al., 2022a). Therefore, the mass transport through porous bone substitutes that is mainly measured by permeability, should be well designed to allow for sufficient oxygenation and delivery of nutrients to residing cells (Karande et al., 2004; Hollister, 2005; Dias et al., 2012; Truscello et al., 2012; Bobbert et al., 2017).

Permeability (k) is a proportionally constant between the average velocity of liquid passing through a porous structure at an applied pressure gradient and is defined by Darcy’s law as presented in Eq. 2 (Zhianmanesh et al., 2019):

$$Ū=-\frac{k}{µ}\nabla p$$ (2)

where Ū shows the average fluid velocity, ∇ P is the applied pressure gradient, and µ is the dynamic viscosity. Several studies have characterized the permeability of porous scaffolds for biomedical applications (Tamayol and Bahrami, 2009). In this way, the constructs with minimal surfaces have received special attention. They have specific geometrical properties that make them appealing for bone tissue regeneration. For these surfaces, the mean curvature is zero which resembles the mean curvature of trabecular bone (Bobbert et al., 2017).

Scaffold pore size is a vital parameter in TE since it promotes cell adhesion, proliferation and differentiation. In this way, modulation of pore size distribution would change the permeability of the TE scaffolds. Al-Munajjed et al. (2008) showed for hyaluronic-collagen scaffolds that the permeability and porosity of scaffolds were increased as pore size was enlarged. Larger pores create less resistance for fluid to pass through the scaffold and provide higher Darcy’s constant (Al-Munajjed et al., 2008).

As for scaffolds with higher permeability, cell suspension experiences less resistance once permeate through the scaffold. This leads to faster stream, which give cells shorter time period to attach to a solid surface. Thus, seeding could be more productive for structures with smaller pores, i.e., lower permeability values (Van Bael et al., 2012).

### 3.3 Effect of porosity on interfacial adhesion and vascularization

In general, pore structure is a significant consideration for TE constructs. Pores must be highly interconnected to allow for cellular, migration and proliferation and diffusion of required substances. Specific surface area per unit mass is an important design parameter that affects the interfacial cell adhesion of a scaffold. For the scaffolds with small pores, formation of cellular capsules around the edges of pores can limit the delivery of nutrients and oxygen (Mostafavi et al., 2021; Lv et al., 2022b; Lv et al., 2022c). On the contrary, too large pores reduce the surface area and limit the cellular adhesion (Murphy et al., 2010).

In a study, Torres-Sanchez et al. (2017) found that small pores enhanced cell attachment and showed higher cell growth rate until the third day of cell culture owing to the larger surface area. However, larger pores supported the cell proliferation and had larger cell growth rate after the third day (Yuan et al., 2019). Due to the importance of surface area on cell adhesion, the upper and lower values of pore size is a major design consideration for collagen scaffold. O’Brien et al. (2005) tested this hypothesis that the level cell attachment is modulated by the average pore size. It was observed that cell binding and activity could be altered significantly affected by the type of cell, as well as composition and pore size of a scaffold. It could be expected that TE of each construct should require an appropriate pore size [O’Brien et al. (2005)].

The second parameter that affects the interfacial adhesion is the surface structure of scaffold substrate. Surface structure of an implant plays a crucial role in biocompatibility, bioactivity and osseointegration of a scaffold. After implantation, surface of a scaffold directly starts to interact with surrounding bio fluids and tissues. Adsorption of proteins is affected by the scaffolds surface texture and chemistry. Scaffold surface chemistry can affected by the roughness of the surface, response to wettability, and its mechanical attributes (O’Brien et al., 2005; Dave and Gomes, 2019). In this way, cellular-related activities including binding, proliferation and differentiation are highly regulated by the composition and type of absorbed proteins on the surface of the scaffold (Civantos et al., 2017).

## 4 Additively manufactured scaffolds

3D printing, also known as additive manufacturing (AM), has shown great potential for bone tissue engineering by enabling the fabrication of customized porous scaffolds that mimic the structural properties of natural bone extracellular matrix (Tables 1, 2) (Praveena et al., 2022). The key 3D printing technologies explored for bone scaffold fabrication can be categorized as follows.

**TABLE 1:** Summary of the composition, printing technique, pore size, and biological effect of different 3D porous composite scaffolds.

Scaffold composition	Bioprinting technique	Pore size	*In vitro*/*In vivo* effect	Ref
Poly (lactic-co-glycolic acid) (PLGA)	FDM	300–700 μm; actual printed scaffolds had 221–775 μm pores	Good cytocompatibility with fibroblasts, increased osteoblast adhesion and proliferation over 7 days	Liu et al. (2018b)
Polypropylene fumarate (PPF), polyethylene glycol-polycaprolactone (PEG-PCL-PEG), pluronic (PF127)	D Bioplotter^®^ using pressure and temperature regulated syringe	600 μm	Sustained release of simvastatin over 20 days, restored mechanical properties of fractured human clavicle bone to 99% matrix hardness and 98% matrix resilience of healthy bone	Kondiah et al. (2020)
PCL	Extrusion-based printing	Pore dimensions on the order of a few hundred microns	Compressive mechanical properties measured and compared between scaffolds with different inner geometries (lattice, wavy, hexagonal, shifted); Finite Element Modeling was employed to predict compressive properties of the scaffolds, Good agreement found between modelled and experimentally measured properties; properties tailored over a range by varying the inner geometry while keeping overall porosity constant	Awwad et al. (2020)
90% attapulgite (ATP) nanorods + 10% polyvinyl alcohol (PVA) binder	3D bioprinting with pneumatic extrusion	500 μm channels but the actual printed scaffolds had 20–50 μm pores	Good biocompatibility with osteoblasts; Increased osteogenic gene expression, More calcium deposition; More bone formation vs. controls in rat model, Bone growth directly on scaffold surface, Increased blood vessel formation	Wang et al. (2020)
Silk fibroin-gelatin composite with cell-laden alginate-collagen core-shell microgels	Extrusion-based 3D bioprinting	—	Microgel-15% silk fibroin/8% gelatin showed highest cell viability compared to scaffolds without microgels/Microgel-15% silk fibroin/8% gelatin showed better bone regeneration compared to 15% silk fibroin/8% gelatin scaffold without microgels	Chai et al. (2021)
Hydroxyapatite (HA) loaded with superparamagnetic iron oxide nanoparticles (SPIONs)	3D bioprinted with a geometry that closely corresponded to the bone defect using a surgically friendly bioink mainly composed of hydroxyapatite	—	*In vitro* culture of mouse embryonic cells and human osteoblast-like cells on the printed HB scaffolds showed viability and functionality for up to 14 days/Implantation of the bioprinted HB scaffolds into a rat model of femoral bone defect demonstrated significant regenerative effects over a 2-week time course. The HB grafts showed rapid integration with host tissue, ossification, and growth of new bone. No infection, immune rejection, or fibrotic encapsulation was observed	Shokouhimehr et al. (2021)
90% PCL+ 10% amorphous calcium phosphate (ACP)	Pneumatic gelling liquid extrusion	50–710 μm	Compressive strength 2–12 MPa, Interconnected pores confirmed by SEM, Repeatable pore structure	Roque et al. (2021)
PCL	Hybrid bioprinting: Fused deposition modeling (FDM) of PCL combined with microextrusion of alginate-gelatin cell-laden hydrogel	0.53–2.92 mm^3^	Printing temperature of 140°C provided good balance between PCL filament bond strength and cell viability in surrounding hydrogel, Compressive modulus of up to 6 MPa achieved for bare PCL scaffolds, decreasing to ∼4 MPa for hybrid PCL-hydrogel constructs, No significant degradation of mechanical properties observed over 28 days incubation of hybrid constructs with encapsulated cells	Koch et al. (2022)
PCL and micron-sized barium titanate (BaTiO3) particles	Extrusion-based 3D printing, specifically fused filament fabrication (FFF)	320 µm	The scaffolds with a mean pore size of 320 µm resulted in the highest pre-osteoblast growth kinetics, Ultrasonic stimulation (US) at 1 Hz enhanced pre-osteoblast adhesion, proliferation, and spreading, Ultrasonic stimulation at 3 Hz benefited osteoblast differentiation by upregulating important osteogenic markers	Sikder et al. (2022)
Void-forming hydrogel prepared by digital light processing (DLP)-based bioprinting of bone marrow stem cells (BMSCs) mixed with gelatin methacrylate (GelMA)/dextran emulsion	Digital light processing (DLP)-based bioprinting	—	The 3D-bioprinted hydrogel promotes the proliferation, migration, and spreading of the encapsulated BMSCs, The porous structure of the hydrogel enhances cell spreading, migration, and proliferation of the encapsulated BMSCs, The niche created by the porous structure stimulates the YAP signal pathway, leading to enhanced osteogenic differentiation of BMSCs, The porous structure of the hydrogel forces YAP nuclear localization and upregulation of YAP targeted genes/The void-forming hydrogel shows great potential for BMSCs delivery and significantly promotes bone regeneration *in vivo*, The generated pores in the 3D-bioprinted hydrogels significantly promote skull repair *in vivo*	Tao et al. (2022)
Cartilage phase: Alginate-gelatin (A-G) hydrogel	Extrusion-based bioprinting of A-G hydrogel/Direct ink writing (DIW) of PCL/HA composite	—	A-G hydrogel supported high viability and proliferation of encapsulated chondrocytes, PCL/HA composite supported attachment, spreading, proliferation, and mineralization of seeded osteoblasts	Chen et al. (2023)
Bone phase: PCL with HA microparticles				
Gelatin methacrylate (GelMA), polyethylene glycol diacrylate (PEGDA), and Pluronic F127 diacrylate (F127DA)	Digital light processing (DLP) printing	—	The GelMA/PEGDA/F127DA (GPF) scaffold facilitated the adhesion and proliferation of cells and promoted the osteogenic differentiation of mesenchymal stem cells in an osteoinductive environment, The osteogenic differentiation of rat bone marrow mesenchymal stem cells (rBMSCs) was not promoted by either the PEGDA/F127DA (PF) or GPF scaffolds/The bone tissue volume	Gao et al. (2023)
Methacrylated gelatin (GelMA)/methacrylated alginate (AlgMA) system, with the addition of rat platelet-rich plasma (PRP) and a nanoclay called laponite (Lap)	layer-by-layer printing of the hydrogel bioink with PCL	—	The PRP-GA@Lap hydrogel significantly promoted the proliferation, migration, and osteogenic differentiation of rat bone marrow mesenchymal stem cells, accelerated the formation of endothelial cell vascular patterns, and promoted macrophage M2 polarization/*In vivo* experiments using subcutaneous and femoral condyle defects in rats showed that the PRP-GA@Lap/PCL scaffolds significantly promoted vascular inward growth and enhanced bone regeneration at the defect site	Gao et al. (2023)
10%–15% gelatin methacryloyl (GelMA)	Microextrusion	—	Bioprinting enhanced osteogenic gene expression compared to 2D culture, 2 weeks pre-induction + 3 weeks post-induction osteogenic culture showed highest osteogenic potential *in vitro* and bone formation *in vivo*, Similar bone formation for 5 weeks total osteogenic induction regardless of pre- vs. post-induction timing, Residual GelMA observed after 8 weeks implantation in rat calvarial defect	Raveendran et al. (2023)
Silk fibroin/gelatin composite scaffold loaded with silicon nitride (Si3N4) nanoparticles	Low-temperature 3D bioprinting	600–700 μm	Good cytocompatibility, Promoted osteogenic differentiation of rat BMSCs/1% Si3N4 scaffold showed best bone regeneration in rat femoral defect model	Yunsheng et al. (2023)
Alginate-HA	Extrusion-based 3D bioprinting	150 μm	Good interconnectivity between pores, 80% porosity	Krishna and Sankar (2023)

**TABLE 2:** Materials employed for 3D printing of tissue engineered bone substitute.

Composition	Features	Reference
Titanium coated with chitosan- hydroxyapatite	Enhanced proliferation, differentiation, and osteogenesis of MC3T3-E1 cells	Wei et al. (2018)
Titanium	Promoting collagen-producing, alkaline phosphatase activities, and osteocalcin level	Maleksaeedi et al. (2013)
Titanium coated with chitosan magnesium calcium silicate	Promoting regeneration of the critical size bone defects	Tsai et al. (2019)
Tricalcium phosphate	Mg^2+^ induces cellular adhesion, proliferation, and alkaline phosphatase expression, Si^4+^ shows stimulatory effect on proliferation, osteogenic, differentiation, and mineralization of preosteoblasts	Bose et al. (2017)
Hydroxyapatite	Improving the ability of ceramic templates to promote bone healing	Dutta Roy et al. (2003)
MgP	MgP is completely degradable at 4 weeks; pore architecture formed by the template struts greatly influences bone formation	Kim et al. (2016)
CaP	It creates a proper combination of growth, cell populations and other osteoinductive elements for bone regeneration	Kruth et al. (2005)
Polycaprolactone-fish bone extract	Supporting cell proliferation, inducing calcium deposition, expression of osteogenic markers such as bone morphogenic protein, osteocalcin, alkaline phosphatase, and osteopontin	Heo et al. (2019)
Poly (propylene fumarate) resin	No sign of inflammation, the formation of lamellar bone bridges in critical-sized cranial defects of a rat model	Nettleton et al. (2019)
Polycaprolactone-bioactiveglass	Proliferation and viability of the fibroblast cells	Korpela et al. (2013)
Polycaprolactone	The cell-seeded constructs revealed about 60% more calcification area than the unseeded templates orunrepaired defects	Jensen et al. (2014)
Ppolydimethylsiloxane (PLGC)	PLGC template with hDPSCs/OF induced highest new bone formation	Kwon et al. (2015)
Polyamide-hydroxyapatite	Supporting cell migration, expression of alkaline phosphatase, accelerate the new femoral bone formation	Ramu et al. (2018)
chitosan-hydroxyapatite	Enhancement of osteoconductivity	Ang et al. (2002)
polycaprolactone mixed with β-tricalcium phosphate	New bone formation 8 weeks after implantation in rabbit calvarial defects	Pae et al. (2019)
Polylactic acid-glycolic acid copolymer/tertiary calcium phosphate	Increasing osteoconductive capacity	Pati et al. (2015)
wollastonite/Magnesium/tertiary calcium phosph -ate (CSi/Mg/TCP)	CSi/Mg/TCP templates showed significant synergetic effect on osteoconductivity than CSi or TCP templates alone	Shao et al. (2017)
Human induced pluripot -ent stem cell-derived cardiomyocytes (hiPSC-CM)	Enabled cost-effective, reproducible and scalable hiPSC- CM production with high activity for tissue engineering, drug screening and regenerative medicine	Sasano et al. (2020)
Alginate, gelatin and human mesenchymal stem cells	Optimizing for stiffness and cell density, showing great promise for bone tissue engineering applications	Zhang et al. (2020)
Collagen-infilled 3D printed scaffolds loaded with miR-148b-transfect -ed bone marrow stem cells	3D printing enabled the fabrication of hybrid scaffolds for calvarial defect repair; miR-148b-transfected stem cells underwent early differentiation in hybrid scaffolds; miR-148b-transfected stem cells improve bone regeneration in rat calvarial defects	Lih et al. (2019)
barium titanate and 45S5 bioactive glass	Piezoelectric properties with piezoelectric constant d33 ranging from 1–21 pC/N, Compressive strength of 23.8–56.4 MPa, Formed hydroxyapatite layer during *in vitro* bioactivity testing, Cytocompatible with pre-osteoblast cells	Polley et al. (2023)
β-TCP and CaSiO3	Interconnected porous architecture fabricated by 3D printing, Coculture system of HUVECs and hBMSCs promoted osteogenesis and angiogenesis, Induced early osteogenic protein secretion and capillary tube formation *in vivo*	Liu et al. (2022)
PCL or PLLA with β-TCP	Produced by fused deposition modeling 3D printing, β-TCP content up to 50 wt%, Rough and porous surface morphology, Young’s modulus around 100–800 MPa, Compressive strength up to 67 MPa, Non-cytotoxic to fibroblast and osteoblast cells	Podgórski et al. (2023)
Bentonite and HA	Fabricated by robocasting 3D printing, Compressive strength up to 52 MPa, Porosity 31%–38% and water absorption 28%–45%, Degradation rate 15%–20% after 28 days, Biocompatible with 91% cell viability	Logeshwaran et al. (2023)
84 wt% HA particles in PCL matrix treated with 2 and 2.5 M NaOH	Improved surface hydrophilicity, reduced foreign body reaction, promoted M2 polarization and bone formation, Excessive corrosion of PCL, rapid degradation, weaker mechanical properties	Li et al. (2023a)

### 4.1 Extrusion-based techniques

Fused deposition modeling (FDM) is a widely used methodology due to its simplicity and cost-effectiveness. It employs a thermoplastic polymer filament that is heated and then extruded layer by layer through a nozzle, allowing for the creation of a desired scaffold structure. FDM allows for the incorporation of ceramic particles into polymer to improve bone bioactivity. However, high temperatures limit direct printing of cells or bioactive factors. Overall, FDM is ideal for rapidly fabricating customized biopolymer bone scaffolds (Lee et al., 2019; Jiang and Ning, 2020; Winarso et al., 2022; Zhang et al., 2023). Numerous 3D porous scaffolds were created using FDM method (Lee et al., 2019; Ferrández-Montero et al., 2020; Jiang and Ning, 2020; Dong et al., 2021; Jiang et al., 2021).

### 4.2 Inkjet printing techniques

Inkjet bioprinting utilizes thermal or piezoelectric mechanisms to eject bioink droplets containing cells, growth factors, and other components onto a platform in order to create tissues. This methodology facilitates the generation of bioactive bone scaffolds with exceptional accuracy. However, there are limitations regarding the consistency of bioinks that can be printed (Jamee et al., 2021; Kumar et al., 2021; Parodi et al., 2023).

Generally, inkjet bioprinting offers the advantages of high output, cost-effectiveness, easy implementation, and compatibility with low viscosity biomaterials (Yenilmez et al., 2019; Dell et al., 2022). Consequently, inkjet bioprinting is extensively used in preclinical research and clinical applications (Lv et al., 2018; Li et al., 2020).

### 4.3 Laser-based techniques

#### 4.3.1 Selective laser sintering (SLS)

SLS is a manufacturing technique that uses a laser to fuse layers of a powdered material based on a three-dimensional (3D) model. The process involves heating and fusing a thin layer of powder using a laser beam that follows a predetermined scanning path. This process is repeated layer by layer until a 3D porous scaffold is formed. The SLS technique has proven to be effective in producing a variety of scaffolds suitable for bone tissue engineering, with an optimal pore structure and improved mechanical properties. This technique has been successfully used to create porous scaffolds using both polymers and metals (Sun et al., 2016; Shuai et al., 2018).

#### 4.3.2 Stereolithography (SLA)

SLA is a 3D printing technique that uses a UV laser to selectively cure and solidify liquid photopolymer resins in layer-by-layer fashion to build a 3D object. This approach possesses a remarkable level of precision and has the capability to generate complex internal configurations. Nonetheless, the range of available materials is limited, and there is a potential hazard associated with the use of toxic resins (Shirvan et al., 2021; Raguraman and Rajan, 2023). Previous researches have reported successful fabrication of 3D scaffolds for bone repair using SLA technique (Elomaa et al., 2020; Ronca et al., 2021).

### 4.4 Low-temperature printing techniques

Low-temperature deposition manufacturing (LDM) utilizes a process of extrusion to avoid the risks of temperature-induced damage to cells and proteins, thereby ensuring their intact wellbeing. Freeze-drying removes solvents from printed parts. LDM can create bone scaffolds with nano-scale pores for cell infiltration. However, harsh solvents are involved, and weaker structures are produced. LDM uniquely facilitates room-temperature printing of hierarchically porous bioactive bone scaffolds (Zerankeshi et al., 2022). The applications of LDM to fabricate 3D porous polymer-metal composite bone scaffolds have been reported (Ma et al., 2020; Long et al., 2021; Ali et al., 2022).

### 4.5 Biological 3D printing

This technique focuses on printing cell-laden hydrogel-based bioinks to generate living bone tissue constructs. Key techniques include inkjet bioprinting, extrusion bioprinting, and laser-assisted bioprinting. These methods allow for the printing of bone constructs encapsulating living cells, growth factors, etc. They are essential for fabricating vascularized, functional bone grafts with a physiological cell distribution (Arastouei et al., 2021; Zhou et al., 2021; Maresca et al., 2023). Among the available techniques, FDM is the most widely used 3D printing technique for bone scaffolds due to its simplicity, low cost, and ability to process a range of biomaterials. However, extrusion-based techniques like FDM provide lower resolution compared to light-based methods like SLA or SLS. Inkjet bioprinting enables high precision cell printing but has limitations on the viscosity of printable. The optimal choice depends on factors such as desired resolution, mechanics, and incorporation of biological components. Each 3D printing approach has its own advantages and disadvantages that make it suitable for different bone tissue engineering applications (Ngo et al., 2018; Gharibshahian et al., 2023).

## 5 Triply periodic minimal surfaces (TPMS)

Triply periodic minimal surface (TPMS) structures have a higher surface-area-to-volume ratio in comparison with conventional lattice structures. Specifically, the TPMS sheet constructs exhibit significantly large surface areas. The advantage of high surface area for TPMS-based scaffolds is an enhancement in cell adhesion, migration and proliferation. Besides, these geometries have an infinitely continued surface with smooth joints that causes lower levels of stress concentration and enhances the mechanical strength of the scaffold. TPMS surfaces known to Schwartz works and defined as periodically infinite structures along with three independent axes that have zero mean curvature of the surface (Karcher, 1989; Yuan et al., 2019; Lv et al., 2022d).

**FIGURE 4:**
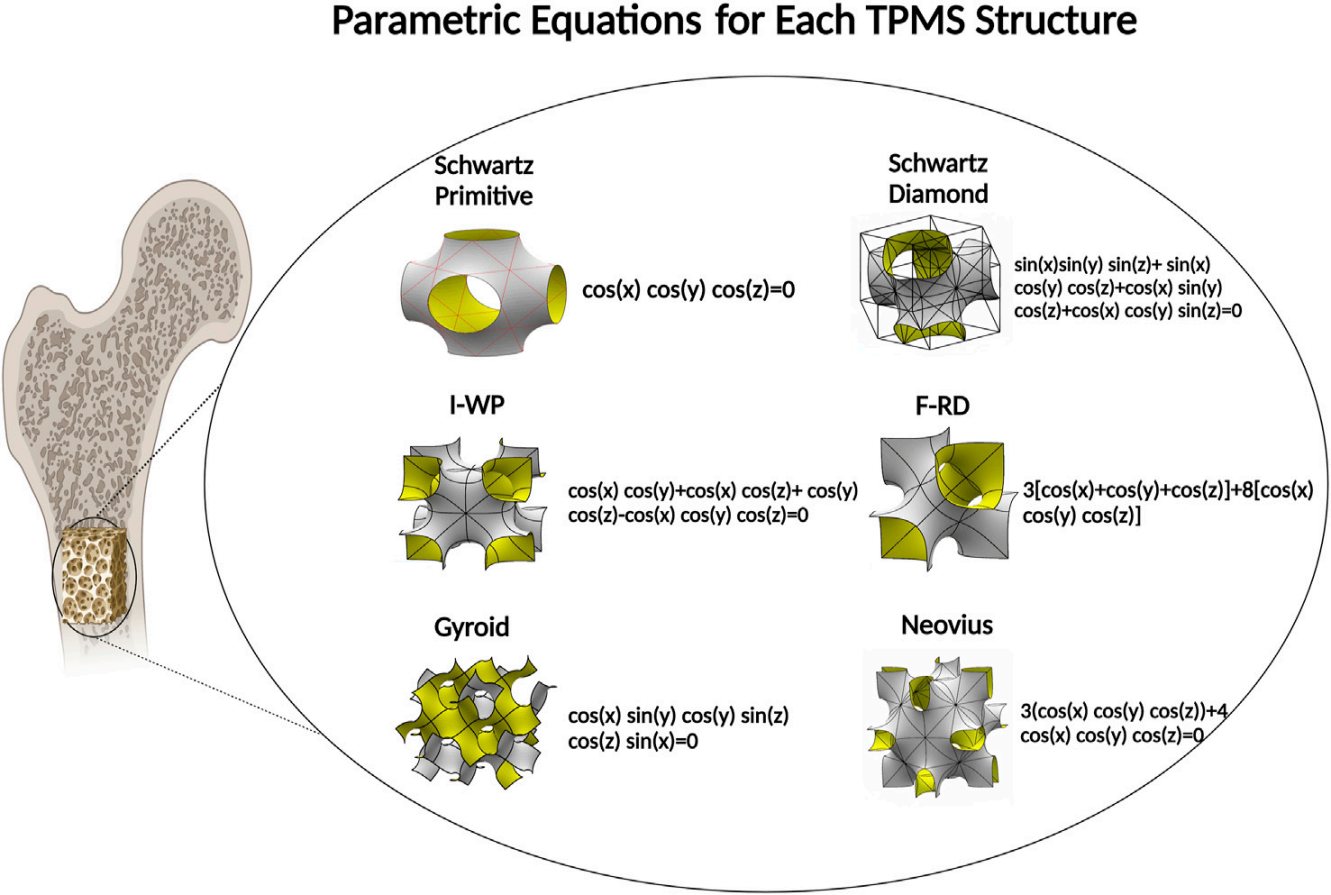
Topological design based on six different types for TPMS porous biomaterial fabrication.

Titanium alloys have been commonly employed as the most appropriate materials for biomedical devices. Such metal alloys have elastic modulus greater than bone tissues that this mismatch in mechanical properties may lead to stress shielding (Claes et al., 2003; Truscello et al., 2012; Liu et al., 2018; Kondiah et al., 2020; Samandari et al., 2022). In this case, stress is removed from bone and majority of exerted forces are bypassed through adjacent implanted scaffolds. Stress shielding results in failure of bone scaffold (Wang et al., 2019) due to the reduction of bone density. A healing bone usually remodels itself at the presence of mechanical loadings. This remodelling process helps bone to adjust its mechanical properties in response to loading. Reduction in load, due to the existence of the implanted scaffold, would cause a bone to become thinner and weaker because there are no stimuli to induce remodelling (Brien et al., 2005). To solve this problem, either a metallurgical method can be applied or porosity can be introduced into the metals (Wang et al., 2019; Yuan et al., 2019). Porosity in metallic constructs reduces the elastic modulus of metal materials which result in stress transfer between bone tissue and its adjacent construct, and allows the porous construct, i.e., scaffold, to get integrated into bone tissue, making long-term osteointegration achievable (Wang et al., 2019). Several studies have been performed to find correlations between design parameters and mechanical properties of porous scaffolds (Bobbert et al., 2017; Maskery et al., 2018; Al-ketan et al., 2019; Wang et al., 2019). Among different methods of fabricating porous structure, the additive manufacturing techniques are prominent because of their abilities in optimization of TPMS structures due to their dimensional and high level of design flexibility with periodic regular structures (Bobbert et al., 2017) (Figure 4). Li et al. (2019) used graded TPMS porous scaffolds and showed that these types of scaffolds are more suitable technique for implant fabrication.

In the last decade, TPMS scaffolds have been fabricated from a variety of materials including metals, polymers (Karageorgiou and Kaplan, 2005; Karageorgiou and Kaplan, 2005; Faramarzi et al., 2018; Martinez-marquez et al., 2018; Cai et al., 2019; Efraim et al., 2019; Ostrovidov et al., 2019; Yan et al., 2019; Gerdes et al., 2020; Javid-Naderi et al., 2023), ceramics (Hulbert et al., 1970; Kokubo, 1996; Will et al., 2004; Zhang et al., 2016; Bobbert et al., 2017; Elsayed et al., 2017; Diaz-Rodriguez et al., 2018; Toosi et al., 2022), as well as hydrogels (Somo et al., 2015; Bianco et al., 2017; Mohammadi et al., 2018; Wong et al., 2019). 3D printed porous scaffolds from metals, polymers, and ceramics offer mechanical properties comparable to native bones. The structural and mass transport properties of these scaffolds, especially permeability, have a significant effect on bone formation and implant integration.

AM has shown major potential for manufacturing complex structures such as TPMS with extremely extending surfaces. Researchers have demonstrated the feasibility of 3D printing TPMS structures for bone regeneration applications. In a study TPMS structures fabricated with 316L stainless steel by using SLM technology, and discovered their mechanical properties and energy absorption capacities (Zhang et al., 2018). In another one, with SLM printed TPMS specimens compare between finite element method and experimental data then examine elastic modulus, yielding strength, stress strain distribution and the failure occurrence mechanisms of Ostrovidov et al. (2019). In other study, Abueidda et al. (2017) used SLS technology to make different TPMS samples and established that different porosity can alter the mechanical properties of structures. Maskery found that the polymer 3D printed Schwarz primitive lattice displayed stretching and bulking, whereas the Gyroid and diamond deformed in a blending manner (Maskery et al., 2018). A list of scaffolds with TPMS structures is provided in Table 3.

**TABLE 3:** Summary of the TPMS structure, substrate materials, printing technique, porosity and mechanical properties.

TPMS structure	Substrate materials	Printing technique	Porosity	Mechanical properties	Ref
Primitive, IWP, Neovius	Polyamide 12	Selective laser sintering	4%–25%	Compressive modulus and strength increase with relative density. Neovius and IWP have higher modulus and strength than Primitive	Abueidda et al. (2017)
Primitive, Diamond, Gyroid	316L stainless steel	Selective laser melting	60%–80%	Diamond has highest compressive modulus. All TPMS structures outperform BCC lattice in stiffness, strength and energy absorption	Zhang et al. (2018)
Gyroid	Wollastonite	Digital light processing	50%–55%	Gyroid structure had lower compressive strength than cubic and cylindrical pore structures	Li et al. (2023b)
Diamond, Gyroid, Primitive, Lattice	Ti6Al4V	SLM	50%–70%	Diamond and s-Diamond had highest compressive strength and elastic modulus	Zhu et al. (2018)
Diamond, Gyroid, Schwarz	PLA	FDM	35%–65%	Elastic modulus: 170–324 MPa, Compressive strength: 5–27 MPa	Diez-Escudero et al. (2020)
Schwarz-P, Gyroid	PLA/Graphene oxide (GO) nanocomposite	FDM	∼50%	Compressive modulus: 60–90 MPa, Compressive strength: 9.8–11.3 MPa	Guo et al. (2023)
Various, including Primitive, Gyroid, Diamond	Various, including polymers, metals, ceramics	Various AM techniques discussed	Variable porosity discussed	Wide range of mechanical properties discussed	Pugliese and Graziosi (2023)
Primitive	Stainless steel	SLM	Designed: 75%–90%	Not studied	Zhu et al. (2022)
Diamond, Gyroid, Primitive	HA	3D printing (CeraFab 7500)	70%–82%	Diamond and Gyroid had ×2 higher compressive strength than Primitive and Lattice; Diamond had highest Young’s modulus	Maevskaia et al. (2023)
I-WP lattice	PLA + 2.5–10% porous iron particles	SLS	40% relative density	2.75–4.28 MPa compressive strength	Xu et al. (2023)
Gyroid	VisiJet M3 Crystal	3D MultiJet printing	50%–70%	Lower porosity correlated with higher stiffness; numerical predictions matched experimental data	Castro et al. (2019)
Schwartz Surface, Diamond, Gyroid	Ti6Al4V	SLM	50%–80%	G had smoothest variation in mechanical properties across porosities; S had steep variation	Lv et al. (2022e)
Diamond, s-Diamond, Gyroid, s-Gyroid, IWP	Wollastonite	Digital light processing	50%–60%	s-Diamond and s-Gyroid had ×3–4 higher compressive strength than Diamond, Gyroid, IWP	Shen et al. (2023)
Primitive, Diamond, Gyroid, Octo	—	Finite element analysis	50%–75%	Anisotropic arrangements matched bone elastic properties. Accuracy within 3% for 3 targets and 5% for 6 targets	Liu et al. (2023a)
Bredigite	Wollastonite	Digital light processing	50%–70%	TPMS structure had significantly better mechanical properties than open-rod scaffold with same porosity	Liu et al. (2023b)
Gyroid, Diamond	Ti6Al4V	Selective laser melting	50%–60%	Elastic modulus 10.6–11.2 GPa. Yield strength 367–419 MPa. Stable properties in different loading directions	Ye et al. (2023)

## 6 Conclusion and future directions

In summary, this paper provides a review on the effect of porosity, pore size, pore structure and interfacial adhesion on the exchange of nutrient, vascularization, and bone formation. Special attention is given to the AM porous structures, especially TPMS scaffolds. The main conclusions are as follows:

(1) Porous structures that facilitate cell differentiation; migration and formation of blood vessels are desirable for implant applications. Porous scaffolds with 200–350 µm pore size, which mimics the porosity of cancellous bone, facilitate bone ingrowth. It is shown that increasing pore size increases permeability and porosity. In addition, *in vivo* experiments suggest that larger pore size enhances vascularization, and higher porosity enhances osteogenesis and bone formation. The specific surface area is another factor that affects osteointegration. It is shown that smaller pore size for a scaffold provides a larger specific surface area that is an important factor for cell attachment. Thus, there is a compromise between the optimum pore size for the cell migration and the specific surface area.
(2) Use of porous metallic scaffolds reduces the effect of stress shielding, which results in stress transfer between bone tissue and scaffolds. This effect results in the integration of porous structure in bone tissue and in making long-term physiological fixation. Among different methods of fabricating porous structures, AM of the TPMS surfaces is the optimal method.
(3) Permeability, which can be considered as an independent design parameter, is an essential parameter in determining the mass transport properties of a scaffold for sufficient delivery of nutrients and proper oxygenation.
(4) Engineering scaffolds with multiscale porosity is challenging and 3D printing is a powerful tool for achieving that. However, advanced biomaterials inks and printing nozzles are needed to be developed to achieve a combination of micro to macropores. Another important consideration is the method for the implantation of scaffolds. The fixation of polymeric and hydrogel scaffolds in place is challenging and therefore new strategies are needed to facilitate the implantation of scaffolds. One emerging technique is *in vivo* 3D (bio) printing, where the scaffold is directly built inside the defect site (Samandari et al., 2022). Such technique eliminates the need to implantation and also facilitate the formation of scaffolds that seamlessly fit the defect site. This area is expected to advance the field of bioprinting for treating complex injuries.
(5) 3D-printed biodegradable metallic scaffolds are also emerging that can facilitate improved bore regeneration. In this way, the incorporation of other materials including polymers/hydrogels into the metallic scaffolds can create a composite structure that can provide proper mechanical strength with suitable microenvironment for cell seeding.
(6) Another critical design consideration is easy handing and manipulation during implantation and surgery for practical use. In this way, scaffold, i.e., bone substitute, should maintain its mechanical shape and integrity during implantation. In addition, any employed materials for scaffold production should enable the conduct of sterilization process prior implanting. Importantly, quality control protocols and regulatory considerations should be also applied to those patient-specific scaffolds and bone substitutes that are produced in factories or hospitals with advanced manufacturing capabilities.

## References

[B1] Abbasi N. Hamlet S. Love R. M. Nguyen N. T. (2020). Porous scaffolds for bone regeneration. J. Sci. Adv. Mater Devices 5 (1), 1–9. 10.1016/j.jsamd.2020.01.007

[B2] Abueidda D. W. Bakir M. Al-Rub R. K. A. Bergström J. S. Sobh N. A. Jasiuk I. (2017). Mechanical properties of 3D printed polymeric cellular materials with triply periodic minimal surface architectures. Mater Des. 122, 255–267. 10.1016/j.matdes.2017.03.018

[B3] Ali D. Sen S. (2017). Finite element analysis of mechanical behavior, permeability and fluid induced wall shear stress of high porosity scaffolds with gyroid and lattice-based architectures. J. Mech. Behav. Biomed. Mater 75, 262–270. 10.1016/j.jmbbm.2017.07.035 28759838

[B4] Ali F. Kalva S. N. Koç M. (2022). Additive manufacturing of polymer/Mg-based composites for porous tissue scaffolds. Polym. (Basel) 14 (24), 5460. 10.3390/polym14245460 PMC9783552 36559829

[B5] Al-ketan O. Lee D. Rowshan R. Al-rub R. K. A. (2019). Functionally graded and multi-morphology sheet TPMS. 10.1016/j.jmbbm.2019.103520 31877523

[B6] Al-Munajjed A. A. Hien M. Kujat R. Gleeson J. P. Hammer’ J. (2008). Influence of pore size on tensile strength, permeability and porosity of hyaluronan-collagen scaffolds. J. Mater Sci. 19, 2859–2864. 10.1007/s10856-008-3422-5 18347950

[B7] Anderson S. B. Lin C.-C. Kuntzler D. V. Anseth K. S. (2011). The performance of human mesenchymal stem cells encapsulated in cell-degradable polymer-peptide hydrogels. Biomaterials 32 (14), 3564–3574. 10.1016/j.biomaterials.2011.01.064 21334063 PMC3085912

[B8] Ang T. H. Sultana F. S. A. Hutmacher D. W. Wong Y. S. Fuh J. Y. H. Mo X. M. (2002). Fabrication of 3D chitosan–hydroxyapatite scaffolds using a robotic dispensing system. Mater Sci. Eng. C 20 (1–2), 35–42. 10.1016/s0928-4931(02)00010-3

[B9] Arastouei M. Khodaei M. Atyabi S. M. Nodoushan M. J. (2021). The in-vitro biological properties of 3D printed poly lactic acid/akermanite composite porous scaffold for bone tissue engineering. Mater Today Commun. 27, 102176. 10.1016/j.mtcomm.2021.102176

[B10] Awwad H. A.-D. M. A. Thiagarajan L. Kanczler J. M. Amer M. H. Bruce G. Lanham S. (2020). Genetically-programmed, mesenchymal stromal cell-laden & mechanically strong 3D bioprinted scaffolds for bone repair. J. Control Release 325, 335–346. 10.1016/j.jconrel.2020.06.035 32629135 PMC7445425

[B11] Bak T.-Y. Kook M.-S. Jung S.-C. Kim B.-H. (2014). Biological effect of gas plasma treatment on CO_ **2** _Gas foaming/salt leaching fabricated porous polycaprolactone scaffolds in bone tissue engineering. J. Nanomater 2014, 1–6. 10.1155/2014/657542

[B12] Bianco S. Mancardi D. Merlino A. Bussolati B. Munaron L. (2017). Hypoxia and hydrogen sulfide differentially affect normal and tumor-derived vascular endothelium. Redox Biol. 12, 499–504. 10.1016/j.redox.2017.03.015 28340463 PMC5369009

[B13] Bobbert F. S. L. Lietaert K. Eftekhari A. A. Pouran B. Ahmadi S. M. Weinans H. (2017). Additively manufactured metallic porous biomaterials based on minimal surfaces: a unique combination of topological, mechanical, and mass transport properties. Acta Biomater. 53, 572–584. 10.1016/j.actbio.2017.02.024 28213101

[B14] Bose S. Tarafder S. Bandyopadhyay A. (2017). Effect of chemistry on osteogenesis and angiogenesis towards bone tissue engineering using 3D printed scaffolds. Ann. Biomed. Eng. 45 (1), 261–272. 10.1007/s10439-016-1646-y 27287311 PMC5149117

[B15] Brien F. J. O. Harley B. A. Yannas I. V. Gibson L. J. (2005). The effect of pore size on cell adhesion in collagen-GAG scaffolds. Biomaterials. 26:433–441. 10.1016/j.biomaterials.2004.02.052 15275817

[B16] Buttery L. D. K. Bishop A. E. (2005). Introduction to tissue engineering. Biomater. Artif. Organs Tissue Eng., 193–200. Chapter 18. 10.1533/9781845690861.4.193

[B17] Cai Z. Liu Z. Hu X. Kuang H. Zhai J. (2019). The effect of porosity on the mechanical properties of 3D-printed triply periodic minimal surface (TPMS) bioscaffold. Bio-Design Manuf. 2 (4), 242–255. 10.1007/s42242-019-00054-7

[B18] Castro A. P. G. Ruben R. B. Gonçalves S. B. Pinheiro J. Guedes J. M. Fernandes P. R. (2019). Numerical and experimental evaluation of TPMS Gyroid scaffolds for bone tissue engineering. Comput. Methods Biomech. Biomed. Engin 22 (6), 567–573. 10.1080/10255842.2019.1569638 30773050

[B19] Chai N. Zhang J. Zhang Q. Du H. He X. Yang J. (2021). Construction of 3D printed constructs based on microfluidic microgel for bone regeneration. Compos Part B Eng. 223, 109100. 10.1016/j.compositesb.2021.109100

[B20] Chen H. Gonnella G. Huang J. Di-Silvio L. (2023). Fabrication of 3D bioprinted Bi-phasic scaffold for bone–cartilage interface regeneration. Biomimetics 8 (1), 87. 10.3390/biomimetics8010087 36975317 PMC10046269

[B21] Chen X. Fan H. Deng X. Wu L. Yi T. Gu L. (2018). Scaffold structural microenvironmental cues to guide tissue regeneration in bone tissue applications. Nanomaterials 8 (11), 960. 10.3390/nano8110960 30469378 PMC6266401

[B22] Cheng M. Wahafu T. Jiang G. Liu W. Qiao Y. Peng X. (2016). A novel open-porous magnesium scaffold with controllable microstructures and properties for bone regeneration. Sci. Rep. 6 (1), 24134–24214. 10.1038/srep24134 27071777 PMC4829853

[B23] Civantos A. Martínez-Campos E. Ramos V. Elvira C. Gallardo A. Abarrategi A. (2017). Titanium coatings and surface modifications: toward clinically useful bioactive implants. ACS Biomater. Sci. Eng. 3 (7), 1245–1261. 10.1021/acsbiomaterials.6b00604 33440513

[B24] Claes L. Eckert-Hübner K. Augat P. (2003). The fracture gap size influences the local vascularization and tissue differentiation in callus healing. Langenbeck’s Arch. Surg. 388 (5), 316–322. 10.1007/s00423-003-0396-0 13680236

[B25] Currey J. D. (1969). The relationship between the stiffness and the mineral content of bone. J. Biomech. 2 (4), 477–480. 10.1016/0021-9290(69)90023-2 16335147

[B26] Daish C. Blanchard R. Pirogova E. Harvie E. Pivonka P. (2019). Numerical calculation of permeability of periodic porous materials: application to periodic arrays of spheres and 3D scaffold microstructures. 1–24. 10.1002/nme.6037

[B27] Dave K. Gomes V. G. (2019). Interactions at scaffold interfaces: effect of surface chemistry, structural attributes and bioaffinity. Mater Sci. Eng. C 105, 110078. 10.1016/j.msec.2019.110078 31546353

[B28] DeLacure M. D. (1994). Physiology of bone healing and bone grafts. Otolaryngol. Clin. North Am. 27 (5), 859–874. 10.1016/s0030-6665(20)30613-7 7816435

[B29] Dell A. C. Wagner G. Own J. Geibel J. P. (2022). 3D bioprinting using hydrogels: cell inks and tissue engineering applications. Pharmaceutics 14 (12), 2596. 10.3390/pharmaceutics14122596 36559090 PMC9784738

[B30] Dias M. R. Fernandes P. R. Guedes J. M. Hollister S. J. (2012). Permeability analysis of scaffolds for bone tissue engineering. J. Biomech. 45 (6), 938–944. 10.1016/j.jbiomech.2012.01.019 22365847

[B31] Diaz-Rodriguez P. Sánchez M. Landin M. (2018). Drug-loaded biomimetic ceramics for tissue engineering. Pharmaceutics 10 (4), 272. 10.3390/pharmaceutics10040272 30551594 PMC6321415

[B32] Diez-Escudero A. Harlin H. Isaksson P. Persson C. (2020). Porous polylactic acid scaffolds for bone regeneration: a study of additively manufactured triply periodic minimal surfaces and their osteogenic potential. J. Tissue Eng. 11, 204173142095654. 10.1177/2041731420956541 PMC7656876 33224463

[B33] Di Luca A. Ostrowska B. Lorenzo-Moldero I. Lepedda A. Swieszkowski W. Van Blitterswijk C. (2016). Gradients in pore size enhance the osteogenic differentiation of human mesenchymal stromal cells in three-dimensional scaffolds. Sci. Rep. 6 (1), 22898–22913. 10.1038/srep22898 26961859 PMC4790631

[B34] Di Martino A. Liverani L. Rainer A. Salvatore G. Trombetta M. Denaro V. (2011). Electrospun scaffolds for bone tissue engineering. Musculoskelet. Surg. 95 (2), 69–80. 10.1007/s12306-011-0097-8 21399976

[B35] Dong Q. Zhang M. Zhou X. Shao Y. Li J. Wang L. (2021). 3D-printed Mg-incorporated PCL-based scaffolds: a promising approach for bone healing. Mater Sci. Eng. C 129, 112372. 10.1016/j.msec.2021.112372 34579891

[B36] Duan B. Wang M. Zhou W. Y. Cheung W. L. Li Z. Y. Lu W. W. (2010). Three-dimensional nanocomposite scaffolds fabricated via selective laser sintering for bone tissue engineering. Acta Biomater. 6 (12), 4495–4505. 10.1016/j.actbio.2010.06.024 20601244

[B37] Düregger K. Trik S. Leonhardt S. Eblenkamp M. (2018). Additive-manufactured microporous polymer membranes for biomedical *in vitro* applications. J. Biomater. Appl. 33 (1), 116–126. 10.1177/0885328218780460 29874967

[B38] Dutta Roy T. Simon J. L. Ricci J. L. Rekow E. D. Thompson V. P. Parsons J. R. (2003). Performance of hydroxyapatite bone repair scaffolds created via three‐dimensional fabrication techniques. J Biomed Mater Res Part A Off J Soc Biomater Jpn. Soc Biomater Aust Soc Biomater Korean Soc Biomater 67 (4), 1228–1237. 10.1002/jbm.a.20034 14624509

[B39] Efraim Y. Schoen B. Zahran S. Davidov T. Vasilyev G. Baruch L. (2019). 3D structure and processing methods direct the biological attributes of ECM-based cardiac scaffolds. Sci. Rep. 9 (1), 5578–5613. 10.1038/s41598-019-41831-9 30944384 PMC6447624

[B40] Einhorn T. A. Gerstenfeld L. C. (2015). Fracture healing: mechanisms and interventions. Nat. Rev. Rheumatol. 11 (1), 45–54. 10.1038/nrrheum.2014.164 25266456 PMC4464690

[B41] Elomaa L. Keshi E. Sauer I. M. Weinhart M. (2020). Development of GelMA/PCL and dECM/PCL resins for 3D printing of acellular *in vitro* tissue scaffolds by stereolithography. Mater Sci. Eng. C 112, 110958. 10.1016/j.msec.2020.110958 32409091

[B42] Elsayed H. Sinico M. Secco M. Zorzi F. Colombo P. Bernardo E. (2017). B-doped hardystonite bioceramics from preceramic polymers and fillers: synthesis and application to foams and 3D-printed scaffolds. J. Eur. Ceram. Soc. 37 (4), 1757–1767. 10.1016/j.jeurceramsoc.2016.12.002

[B43] Faramarzi N. Yazdi I. K. Nabavinia M. Gemma A. Fanelli A. Caizzone A. (2018). Patient-specific bioinks for 3D bioprinting of tissue engineering scaffolds. Adv. Healthc. Mater 7 (11), 1701347. 10.1002/adhm.201701347 PMC6422175 29663706

[B44] Ferrández-Montero A. Lieblich M. Benavente R. González-Carrasco J. L. Ferrari B. (2020). Study of the matrix-filler interface in PLA/Mg composites manufactured by Material Extrusion using a colloidal feedstock. Addit. Manuf. 33, 101142. 10.1016/j.addma.2020.101142

[B45] Gao J. Li M. Cheng J. Liu X. Liu Z. Liu J. (2023). 3D-Printed GelMA/PEGDA/F127DA scaffolds for bone regeneration. J. Funct. Biomater. 14 (2), 96. 10.3390/jfb14020096 36826895 PMC9962173

[B46] Gerdes S. Mostafavi A. Ramesh S. Memic A. Rivero I. V. Rao P. (2020). Process–structure–quality relationships of three-dimensional printed poly (caprolactone)-hydroxyapatite Scaffolds. Tissue Eng. Part A 26 (5–6), 279–291. 10.1089/ten.tea.2019.0237 31964254 PMC7366318

[B47] Gharibshahian M. Salehi M. Beheshtizadeh N. Kamalabadi-Farahani M. Atashi A. Nourbakhsh M.-S. (2023). Recent advances on 3D-printed PCL-based composite scaffolds for bone tissue engineering. Front. Bioeng. Biotechnol. 11, 1168504. 10.3389/fbioe.2023.1168504 37469447 PMC10353441

[B48] Ghelich P. Kazemzadeh-Narbat M. Hassani Najafabadi A. Samandari M. Memić A. Tamayol A. (2022). (Bio)manufactured solutions for treatment of bone defects with emphasis on US-FDA regulatory science perspective. Adv. NanoBiomed Res. 2 (4), 2100073. 10.1002/anbr.202100073 35935166 PMC9355310

[B49] Gugala Z. Gogolewski S. (2002). Healing of critical-size segmental bone defects in the sheep tibiae using bioresorbable polylactide membranes. Injury 33, 71–76. 10.1016/s0020-1383(02)00135-3 12161322

[B50] Guo W. Yang Y. Liu C. Bu W. Guo F. Li J. (2023). 3D printed TPMS structural PLA/GO scaffold: process parameter optimization, porous structure, mechanical and biological properties. J. Mech. Behav. Biomed. Mater 142, 105848. 10.1016/j.jmbbm.2023.105848 37099921

[B51] Henkel J. Woodruff M. A. Epari D. R. Steck R. Glatt V. Dickinson I. C. (2013). Bone regeneration based on tissue engineering conceptions—a 21st century perspective. Bone Res. 1 (1), 216–248. 10.4248/br201303002 26273505 PMC4472104

[B52] Heo S. Ko S. Oh G. Kim N. Choi I. Park W. S. (2019). Fabrication and characterization of the 3D‐printed polycaprolactone/fish bone extract scaffolds for bone tissue regeneration. J. Biomed. Mater Res. Part B Appl. Biomater. 107 (6), 1937–1944. 10.1002/jbm.b.34286 30508311

[B53] Hollister S. J. (2005). Porous scaffold design for tissue engineering. Nat. Mat. 4 (July), 518–524. 10.1038/nmat1421 16003400

[B54] Hollister S. J. Murphy W. L. (2011). Scaffold translation: barriers between concept and clinic. Tissue Eng. - Part B Rev. 17 (6), 459–474. 10.1089/ten.teb.2011.0251 21902613 PMC3223015

[B55] Hulbert S. F. Young F. A. Mathews R. S. Klawitter J. J. Talbert C. D. Stelling F. H. (1970). Potential of ceramic materials as permanently implantable skeletal prostheses. J. Biomed. Mater Res. 4 (3), 433–456. 10.1002/jbm.820040309 5469185

[B56] Hutmacher D. W. (2000). Scaffolds in tissue engineering bone and cartilage. Biomaterials 21 (24), 2529–2543. 10.1016/s0142-9612(00)00121-6 11071603

[B57] Iviglia G. Kargozar S. Baino F. (2019). Biomaterials, current strategies, and novel nano-technological approaches for periodontal regeneration. J. Funct. Biomater. 10 (1), 3. 10.3390/jfb10010003 30609698 PMC6463184

[B58] Jamee R. Araf Y. Naser I. B. Promon S. K. (2021). The promising rise of bioprinting in revolutionalizing medical science: advances and possibilities. Regen. Ther. 18, 133–145. 10.1016/j.reth.2021.05.006 34189195 PMC8213915

[B59] Jang J.-H. Castano O. Kim H.-W. (2009). Electrospun materials as potential platforms for bone tissue engineering. Adv. Drug Deliv. Rev. 61 (12), 1065–1083. 10.1016/j.addr.2009.07.008 19646493

[B60] Javid-Naderi M. J. Behravan J. Karimi-Hajishohreh N. Toosi S. (2023). Synthetic polymers as bone engineering scaffold. Polym. Adv. Technol. 34, 2083–2096. 10.1002/pat.6046

[B61] Jensen J. Rölfing J. H. D. Svend Le D. Q. Kristiansen A. A. Nygaard J. V. Hokland L. B. (2014). Surface‐modified functionalized polycaprolactone scaffolds for bone repair: *in vitro* and *in vivo* experiments. J. Biomed. Mater Res. Part A 102 (9), 2993–3003. 10.1002/jbm.a.34970 24123983

[B62] Jiang D. Ning F. (2020). Fused filament fabrication of biodegradable PLA/316L composite scaffolds: effects of metal particle content. Procedia Manuf. 48, 755–762. 10.1016/j.promfg.2020.05.110

[B63] Jiang D. Ning F. Wang Y. (2021). Additive manufacturing of biodegradable iron-based particle reinforced polylactic acid composite scaffolds for tissue engineering. J. Mater Process Technol. 289, 116952. 10.1016/j.jmatprotec.2020.116952

[B64] Karageorgiou V. Kaplan D. (2005). Porosity of 3D biomaterial scaffolds and osteogenesis. Biomaterials 26 (27), 5474–5491. 10.1016/j.biomaterials.2005.02.002 15860204

[B65] Karande T. S. Ong J. L. Agrawal C. M. (2004). Diffusion in musculoskeletal tissue engineering scaffolds: design issues related to porosity, permeability, architecture, and nutrient mixing. Ann. Biomed. Eng. 32 (12), 1728–1743. 10.1007/s10439-004-7825-2 15675684

[B66] Karcher H. (1989). The triply periodic minimal surfaces of Alan Schoen and their constant mean curvature companions. Manuscripta Math. 64 (3), 291–357. 10.1007/bf01165824

[B67] Kemppainen J. M. Hollister S. J. (2010). Differential effects of designed scaffold permeability on chondrogenesis by chondrocytes and bone marrow stromal cells. Biomater. 31 (2), 279–287. 10.1016/j.biomaterials.2009.09.041 19818489

[B68] Khan S. N. Cammisa F. P. Jr Sandhu H. S. Diwan A. D. Girardi F. P. Lane J. M. (2005). The biology of bone grafting. JAAOS-Journal Am. Acad. Orthop. Surg. 13 (1), 77–86. 10.5435/00124635-200501000-00010 15712985

[B69] Kim J.-A. Lim J. Naren R. Yun H. Park E. K. (2016). Effect of the biodegradation rate controlled by pore structures in magnesium phosphate ceramic scaffolds on bone tissue regeneration *in vivo* . Acta Biomater. 44, 155–167. 10.1016/j.actbio.2016.08.039 27554019

[B70] Kimmel D. B. (1993). A paradigm for skeletal strength homeostasis. J. Bone Min. Res. 8 (S2), S515–S522. 10.1002/jbmr.5650081317 8122521

[B71] Koch F. Thaden O. Conrad S. Tröndle K. Finkenzeller G. Zengerle R. (2022). Mechanical properties of polycaprolactone (PCL) scaffolds for hybrid 3D-bioprinting with alginate-gelatin hydrogel. J. Mech. Behav. Biomed. Mater 130, 105219. 10.1016/j.jmbbm.2022.105219 35413680

[B72] Kokubo T. (1996). Formation of biologically active bone-like apatite on metals and polymers by a biomimetic process. Thermochim. Acta 280–281, 479–490. 10.1016/0040-6031(95)02784-x

[B73] Kondiah P. J. Kondiah P. P. D. Choonara Y. E. Marimuthu T. Pillay V. (2020). A 3D bioprinted pseudo-bone drug delivery scaffold for bone tissue engineering. Pharmaceutics 12 (2), 166. 10.3390/pharmaceutics12020166 32079221 PMC7076403

[B74] Korpela J. Kokkari A. Korhonen H. Malin M. Närhi T. Seppälä J. (2013). Biodegradable and bioactive porous scaffold structures prepared using fused deposition modeling. J. Biomed. Mater Res. Part B Appl. Biomater. 101 (4), 610–619. 10.1002/jbm.b.32863 23281260

[B75] Krishna D. V. Sankar M. R. (2023). Extrusion based bioprinting of alginate based multicomponent hydrogels for tissue regeneration applications: state of the art. Mater Today Commun. 35, 105696. 10.1016/j.mtcomm.2023.105696

[B76] Kruth J.-P. Vandenbroucke B. Van Vaerenbergh J. Mercelis P. (2005). “Benchmarking of different SLS/SLM processes as rapid manufacturing techniques,” in Proceedings of the international conference polymers & moulds innovations PMI, 2005.

[B77] Kumar P. Ebbens S. Zhao X. (2021). Inkjet printing of mammalian cells–Theory and applications. Bioprinting 23, e00157. 10.1016/j.bprint.2021.e00157

[B78] Kwon D. Y. Kwon J. S. Park S. H. Park J. H. Jang S. H. Yin X. Y. (2015). A computer-designed scaffold for bone regeneration within cranial defect using human dental pulp stem cells. Sci. Rep. 5 (1), 12721–12816. 10.1038/srep12721 26234712 PMC4522608

[B79] Lee F. Y. Choi Y. W. Behrens F. F. DeFouw D. O. Einhorn T. A. (1998). Programmed removal of chondrocytes during endochondral fracture healing. J. Orthop. Res. 16 (1), 144–150. 10.1002/jor.1100160124 9565087

[B80] Lee J. Lee H. Cheon K.-H. Park C. Jang T.-S. Kim H.-E. (2019). Fabrication of poly (lactic acid)/Ti composite scaffolds with enhanced mechanical properties and biocompatibility via fused filament fabrication (FFF)–based 3D printing. Addit. Manuf. 30, 100883. 10.1016/j.addma.2019.100883

[B81] Léon Y. León C. A. (1998). New perspectives in mercury porosimetry. Adv. Colloid Interface Sci. 76–77, 341–372. 10.1016/s0001-8686(98)00052-9

[B82] Li L. Shi J. Zhang K. Yang L. Jiang Q. ScienceDirect Early osteointegration evaluation of porous Ti6Al4V scaffolds designed based on triply periodic minimal surface models. 2019;(xxxx). 10.1016/j.jot.2019.03.003 PMC6896722 31844617

[B83] Li W. Xu F. Dai F. Deng T. Ai Y. Xu Z. (2023a). Hydrophilic surface-modified 3D printed flexible scaffolds with high ceramic particle concentrations for immunopolarization-regulation and bone regeneration. Biomater. Sci. 11 (11), 3976–3997. 10.1039/d3bm00362k 37115001

[B84] Li X. Liu B. Pei B. Chen J. Zhou D. Peng J. (2020). Inkjet bioprinting of biomaterials. Chem. Rev. 120 (19), 10793–10833. 10.1021/acs.chemrev.0c00008 32902959

[B85] Li Y. Li J. Jiang S. Zhong C. Zhao C. Jiao Y. (2023b). The design of strut/TPMS-based pore geometries in bioceramic scaffolds guiding osteogenesis and angiogenesis in bone regeneration. Mater Today Bio 20, 100667. 10.1016/j.mtbio.2023.100667 PMC10238647 37273795

[B86] Liao S. Murugan R. Chan C. K. Ramakrishna S. (2008). Processing nanoengineered scaffolds through electrospinning and mineralization suitable for biomimetic bone tissue engineering. J. Mech. Behav. Biomed. Mater 1 (3), 252–260. 10.1016/j.jmbbm.2008.01.007 19627790

[B87] Lih E. Park W. Park K. W. Chun S. Y. Kim H. Joung Y. K. (2019). A bioinspired scaffold with anti-inflammatory magnesium hydroxide and decellularized extracellular matrix for renal tissue regeneration. ACS Cent. Sci. 5 (3), 458–467. 10.1021/acscentsci.8b00812 30937373 PMC6439446

[B88] Lim T. C. Chian K. S. Leong K. F. (2010). Cryogenic prototyping of chitosan scaffolds with controlled micro and macro architecture and their effect on *in vivo* neo-vascularization and cellular infiltration. J. Biomed. Mater Res. Part A 94 (4), 1303–1311. 10.1002/jbm.a.32747 20694998

[B89] Lin W. Chen M. Qu T. Li J. Man Y. (2020). Three‐dimensional electrospun nanofibrous scaffolds for bone tissue engineering. J. Biomed. Mater Res. Part B Appl. Biomater. 108 (4), 1311–1321. 10.1002/jbm.b.34479 31436374

[B90] Lindsey R. W. Gugala Z. Milne E. Sun M. Gannon F. H. Latta L. L. (2006). The efficacy of cylindrical titanium mesh cage for the reconstruction of a critical‐size canine segmental femoral diaphyseal defect. J. Orthop. Res. 24 (7), 1438–1453. 10.1002/jor.20154 16732617

[B91] Lipowiecki M. Ryvolova M. Töttösi Á. Kolmer N. Naher S. Brennan S. A. (2014). Permeability of rapid prototyped artificial bone scaffold structures. J. Biomed. Mater Res. Part A 102 (11), 4127–4135. 10.1002/jbm.a.35084 24443032

[B92] Liu C.-G. Zeng Y.-T. Kankala R. K. Zhang S.-S. Chen A.-Z. Wang S.-B. (2018b). Characterization and preliminary biological evaluation of 3D-printed porous scaffolds for engineering bone tissues. Mater. (Basel) 11 (10), 1832. 10.3390/ma11101832 PMC6213437 30261642

[B93] Liu D. Zhou X. Wang F. Feng Y. Shi Y. (2023b). Research and analysis of the properties of bredigite-based 3D-printed bone scaffolds. Int. J. Bioprinting 9 (3), 708. 10.18063/ijb.708 PMC10236332 37273998

[B94] Liu J. Chen G. Xu H. Hu K. Sun J. Liu M. (2018a). Pre-vascularization in fibrin Gel/PLGA microsphere scaffolds designed for bone regeneration. NPG Asia Mater 10 (8), 827–839. 10.1038/s41427-018-0076-8

[B95] Liu W. Zhang Y. Lyu Y. Bosiakov S. Liu Y. (2023a). Inverse design of anisotropic bone scaffold based on machine learning and regenerative genetic algorithm. Front. Bioeng. Biotechnol. 11, 1241151. 10.3389/fbioe.2023.1241151 37744255 PMC10512832

[B96] Liu X. Ma P. X. (2004). Polymeric scaffolds for bone tissue engineering. Ann. Biomed. Eng. 32 (3), 477–486. 10.1023/b:abme.0000017544.36001.8e 15095822

[B97] Liu X. Zhao N. Liang H. Tan B. Huang F. Hu H. (2022). Bone tissue engineering scaffolds with HUVECs/hBMSCs cocultured on 3D-printed composite bioactive ceramic scaffolds promoted osteogenesis/angiogenesis. J. Orthop. Transl. 37, 152–162. 10.1016/j.jot.2022.10.008 PMC9640992 36380884

[B98] Logeshwaran A. Elsen R. Nayak S. (2023). Mechanical and biological characteristics of 3D fabricated clay mineral and bioceramic composite scaffold for bone tissue applications. J. Mech. Behav. Biomed. Mater 138, 105633. 10.1016/j.jmbbm.2022.105633 36603527

[B99] Long J. Zhang W. Chen Y. Teng B. Liu B. Li H. (2021). Multifunctional magnesium incorporated scaffolds by 3D-Printing for comprehensive postsurgical management of osteosarcoma. Biomaterials 275, 120950. 10.1016/j.biomaterials.2021.120950 34119886

[B100] Lv C. F. Zhu L. Y. Shi J. P. Li Z. A. Tang W. L. Liu T. T. (2018). “The fabrication of tissue engineering scaffolds by inkjet printing technology,” in Materials science forum (Trans Tech Publ), 129–133.

[B101] Lv J. Jin W. Liu W. Qin X. Feng Y. Bai J. (2022e). Selective laser melting fabrication of porous Ti6Al4V scaffolds with triply periodic minimal surface architectures: structural features, cytocompatibility, and osteogenesis. Front. Bioeng. Biotechnol. 10, 899531. 10.3389/fbioe.2022.899531 35694229 PMC9178116

[B102] Lv Y. Guo J. Huang W. Liu Y. Liu W. Wei G. (2022c). Study on bioactivity of SLMed variable gradient TC4 biomedical porous scaffolds with micro-arc oxidation treatment. Anti-Corrosion Methods Mat. 69 (6), 660–666. 10.1108/acmm-08-2022-2684

[B103] Lv Y. Guo J. Zhang Q. Wei G. Yu H. (2022a). Design of low elastic modulus and high strength TC4 porous scaffolds via new variable gradient strategies. Mater Lett. 325, 132616. 10.1016/j.matlet.2022.132616

[B104] Lv Y. Liu G. Wang B. Tang Y. Lin Z. Liu J. (2022b). Pore strategy design of a novel NiTi-Nb biomedical porous scaffold based on a triply periodic minimal surface. Front. Bioeng. Biotechnol. 10, 910475. 10.3389/fbioe.2022.910475 35757802 PMC9214207

[B105] Lv Y. Wang B. Liu G. Tang Y. Liu J. Wei G. (2022d). Design of bone-like continuous gradient porous scaffold based on triply periodic minimal surfaces. J. Mater Res. Technol. 21, 3650–3665. 10.1016/j.jmrt.2022.10.160

[B106] Lv Y. Wang B. Liu G. Tang Y. Lu E. Xie K. (2021). Metal material, properties and design methods of porous biomedical scaffolds for additive manufacturing: a review. Front. Bioeng. Biotechnol. 9, 641130. 10.3389/fbioe.2021.641130 33842445 PMC8033174

[B107] Ma R. Wang W. Yang P. Wang C. Guo D. Wang K. (2020). *In vitro* antibacterial activity and cytocompatibility of magnesium-incorporated poly (lactide-co-glycolic acid) scaffolds. Biomed. Eng. Online 19 (1), 12. 10.1186/s12938-020-0755-x 32070352 PMC7029519

[B108] Maevskaia E. Guerrero J. Ghayor C. Bhattacharya I. Weber F. E. (2023). Triply periodic minimal surface-based scaffolds for bone tissue engineering: a mechanical, *in vitro* and *in vivo* study. Tissue Eng. Part A 29, 507–517. 10.1089/ten.tea.2023.0033 37212290 PMC10611970

[B109] Maleksaeedi S. Wang J. K. El-Hajje A. Harb L. Guneta V. He Z. (2013). Toward 3D printed bioactive titanium scaffolds with bimodal pore size distribution for bone ingrowth. Procedia Cirp 5, 158–163. 10.1016/j.procir.2013.01.032

[B110] Maresca J. A. DeMel D. C. Wagner G. A. Haase C. Geibel J. P. (2023). Three-dimensional bioprinting applications for bone tissue engineering. Cells 12 (9), 1230. 10.3390/cells12091230 37174630 PMC10177443

[B111] Martin T. J. Raisz L. G. Bilezikian J. P. (2008). Principles of bone biology: two-volume set. Academic Press.

[B112] Martinez-marquez D. Mirnajafizadeh A. Carty C. P. Stewart A. (2018). Application of quality by design for 3D printed bone prostheses and scaffolds. PLoS One. 13(4):e0195291. 10.1371/journal.pone.0195291 29649231 PMC5896968

[B113] Maskery I. Sturm L. Aremu A. O. Panesar A. Williams C. B. Tuck C. J. (2018). Insights into the mechanical properties of several triply periodic minimal surface lattice structures made by polymer additive manufacturing. Polym. Guildf. 152, 62–71. 10.1016/j.polymer.2017.11.049

[B114] Maydanshahi M. R. Nazarian A. Eygendaal D. Ebrahimzadeh M. H. Kachooei A. R. Shaegh S. A. M. (2021). 3D printing-assisted fabrication of a patient-specific antibacterial radial head prosthesis with high periprosthetic bone preservation. Biomed. Mater 16 (3), 035027. 10.1088/1748-605x/abe217 33524959

[B115] Mohammadi M. Mousavi Shaegh S. A. Alibolandi M. Ebrahimzadeh M. H. Tamayol A. Jaafari M. R. (2018). Micro and nanotechnologies for bone regeneration: recent advances and emerging designs. J. Control Release 274, 35–55. 10.1016/j.jconrel.2018.01.032 29410062

[B116] Montazerian H. Zhianmanesh M. Davoodi E. Milani A. S. Hoorfar M. (2017). Longitudinal and radial permeability analysis of additively manufactured porous scaffolds: effect of pore shape and porosity. Mater Des. 122, 146–156. 10.1016/j.matdes.2017.03.006

[B117] Mostafavi A. Samandari M. Karvar M. Ghovvati M. Endo Y. Sinha I. (2021). Colloidal multiscale porous adhesive (bio) inks facilitate scaffold integration. Appl. Phys. Rev. 8 (4), 041415. 10.1063/5.0062823 34970378 PMC8686691

[B118] Mota C. Puppi D. Chiellini F. Chiellini E. (2015). Additive manufacturing techniques for the production of tissue engineering constructs. J. Tissue Eng. Regen. Med. 9 (3), 174–190. 10.1002/term.1635 23172792

[B119] Mukherjee S. Darzi S. Paul K. Werkmeister J. A. Gargett C. E. (2019). Mesenchymal stem cell-based bioengineered constructs: foreign body response, cross-talk with macrophages and impact of biomaterial design strategies for pelvic floor disorders. Interface Focus 9 (4), 20180089. 10.1098/rsfs.2018.0089 31263531 PMC6597526

[B120] Murphy C. M. Haugh M. G. O’Brien F. J. (2010). The effect of mean pore size on cell attachment, proliferation and migration in collagen–glycosaminoglycan scaffolds for bone tissue engineering. Biomaterials 31 (3), 461–466. 10.1016/j.biomaterials.2009.09.063 19819008

[B121] Nettleton K. Luong D. Kleinfehn A. P. Savariau L. Premanandan C. Becker M. L. (2019). Molecular mass‐dependent resorption and bone regeneration of 3D printed PPF scaffolds in a critical‐sized rat cranial defect model. Adv. Healthc. Mater 8 (17), 1900646. 10.1002/adhm.201900646 31328402

[B122] Ngo T. D. Kashani A. Imbalzano G. Nguyen K. T. Q. Hui D. (2018). Additive manufacturing (3D printing): a review of materials, methods, applications and challenges. Compos Part B Eng. 143, 172–196. 10.1016/j.compositesb.2018.02.012

[B123] O’Brien F. J. Harley B. A. Yannas I. V. Gibson L. J. (2005). The effect of pore size on cell adhesion in collagen-GAG scaffolds. Biomaterials 26 (4), 433–441. 10.1016/j.biomaterials.2004.02.052 15275817

[B124] Ostrovidov S. Salehi S. Costantini M. Suthiwanich K. Ebrahimi M. Sadeghian R. B. (2019). 3D bioprinting in skeletal muscle tissue engineering. Small 15 (24), 1805530. 10.1002/smll.201805530 PMC6570559 31012262

[B125] Pae H. Kang J. Cha J. Lee J. Paik J. Jung U. (2019). 3D-printed polycaprolactone scaffold mixed with β‐tricalcium phosphate as a bone regenerative material in rabbit calvarial defects. J. Biomed. Mater Res. Part B Appl. Biomater. 107 (4), 1254–1263. 10.1002/jbm.b.34218 30300967

[B126] Parodi I. Di Lisa D. Pastorino L. Scaglione S. Fato M. M. (2023). 3D bioprinting as a powerful technique for recreating the tumor microenvironment. Gels 9 (6), 482. 10.3390/gels9060482 37367152 PMC10298394

[B127] Pati F. Song T. H. Rijal G. Jang J. Kim S. W. Cho D. W. (2015). Ornamenting 3D printed scaffolds with cell-laid extracellular matrix for bone tissue regeneration. Biomater. 37, 230–241. 10.1016/j.biomaterials.2014.10.012 25453953

[B128] Pennella F. Cerino G. Massai D. Gallo D. D’Urso Labate F. G. Schiavi A. (2013). A survey of methods for the evaluation of tissue engineering scaffold permeability. Ann. Biomed. Eng. 41 (10), 2027–2041. 10.1007/s10439-013-0815-5 23612914

[B129] Podgórski R. Wojasiński M. Trepkowska-Mejer E. Ciach T. (2023). A simple and fast method for screening production of polymer-ceramic filaments for bone implant printing using commercial fused deposition modelling 3D printers. Biomater. Adv. 146, 213317. 10.1016/j.bioadv.2023.213317 36738523

[B130] Polley C. Distler T. Scheufler C. Detsch R. Lund H. Springer A. (2023). 3D printing of piezoelectric and bioactive barium titanate-bioactive glass scaffolds for bone tissue engineering. Mater Today Bio 21, 100719. 10.1016/j.mtbio.2023.100719 PMC10387613 37529217

[B131] Poursamar S. A. Lehner A. N. Azami M. Ebrahimi-Barough S. Samadikuchaksaraei A. Antunes A. P. M. (2016). The effects of crosslinkers on physical, mechanical, and cytotoxic properties of gelatin sponge prepared via *in-situ* gas foaming method as a tissue engineering scaffold. Mater Sci. Eng. C 63, 1–9. 10.1016/j.msec.2016.02.034 27040189

[B132] Prabhakaran M. P. Venugopal J. Ramakrishna S. (2009). Electrospun nanostructured scaffolds for bone tissue engineering. Acta Biomater. 5 (8), 2884–2893. 10.1016/j.actbio.2009.05.007 19447211

[B133] Praveena B. A. Lokesh N. Buradi A. Santhosh N. Praveena B. L. Vignesh R. (2022). A comprehensive review of emerging additive manufacturing (3D printing technology): methods, materials, applications, challenges, trends and future potential. Mater Today Proc. 52, 1309–1313. 10.1016/j.matpr.2021.11.059

[B134] Pugliese R. Graziosi S. (2023). Biomimetic scaffolds using triply periodic minimal surface-based porous structures for biomedical applications. SLAS Technol. 28 (3), 165–182. 10.1016/j.slast.2023.04.004 37127136

[B135] Puppi D. Chiellini F. Piras A. M. Chiellini E. (2010). Polymeric materials for bone and cartilage repair. Prog. Polym. Sci. 35 (4), 403–440. 10.1016/j.progpolymsci.2010.01.006

[B136] Raguraman M. Rajan M. (2023). “Nanoengineering/technology for tissue engineering and organ printing,” in Emerging nanotechnologies for medical applications (Elsevier), 35–54.

[B137] Rahbari A. Montazerian H. Davoodi E. Homayoonfar S. (2017). Predicting permeability of regular tissue engineering scaffolds: scaling analysis of pore architecture, scaffold length, and fluid flow rate effects. Comput. Methods Biomech. Biomed. Engin 20 (3), 231–241. 10.1080/10255842.2016.1215436 27494073

[B138] Ramu M. Ananthasubramanian M. Kumaresan T. Gandhinathan R. Jothi S. (2018). Optimization of the configuration of porous bone scaffolds made of Polyamide/Hydroxyapatite composites using Selective Laser Sintering for tissue engineering applications. Biomed. Mater Eng. 29 (6), 739–755. 10.3233/bme-181020 30282331

[B139] Raveendran N. Ivanovski S. Vaquette C. (2023). The effect of culture conditions on the bone regeneration potential of osteoblast-laden 3D bioprinted constructs. Acta Biomater. 156, 190–201. 10.1016/j.actbio.2022.09.042 36155098

[B140] Reichert J. C. Saifzadeh S. Wullschleger M. E. Epari D. R. Schütz M. A. Duda G. N. (2009). The challenge of establishing preclinical models for segmental bone defect research. Biomaterials 30 (12), 2149–2163. 10.1016/j.biomaterials.2008.12.050 19211141

[B141] Ronca A. Ronca S. Forte G. Ambrosio L. (2021). Synthesis of an UV-curable divinyl-fumarate poly-ε-caprolactone for stereolithography applications. Comput. Tissue Eng. Methods Protoc. 2147, 55–62. 10.1007/978-1-0716-0611-7_5 32840810

[B142] Roosa S. M. M. Kemppainen J. M. Moffitt E. N. Krebsbach P. H. Hollister S. J. (2010). The pore size of polycaprolactone scaffolds has limited influence on bone regeneration in an *in vivo* model. J. Biomed. Mater Res. Part A 92 (1), 359–368. 10.1002/jbm.a.32381 19189391

[B143] Roque R. Barbosa G. F. Guastaldi A. C. (2021). Design and 3D bioprinting of interconnected porous scaffolds for bone regeneration. An additive manufacturing approach. J. Manuf. Process 64, 655–663. 10.1016/j.jmapro.2021.01.057

[B144] Roseti L. Parisi V. Petretta M. Cavallo C. Desando G. Bartolotti I. (2017). Scaffolds for bone tissue engineering: state of the art and new perspectives. Mater Sci. Eng. C 78, 1246–1262. 10.1016/j.msec.2017.05.017 28575964

[B145] Rosso F. Marino G. Giordano A. Barbarisi M. Parmeggiani D. Barbarisi A. (2005). Smart materials as scaffolds for tissue engineering. J. Cell Physiol. 203 (3), 465–470. 10.1002/jcp.20270 15744740

[B146] Salerno A. Di Maio E. Iannace S. Netti P. A. (2012). Tailoring the pore structure of PCL scaffolds for tissue engineering prepared via gas foaming of multi-phase blends. J. Porous Mater 19 (2), 181–188. 10.1007/s10934-011-9458-9

[B147] Samandari M. Mostafavi A. Quint J. Memić A. Tamayol A. (2022). *In situ* bioprinting: intraoperative implementation of regenerative medicine. Trends Biotechnol. 40, 1229–1247. 10.1016/j.tibtech.2022.03.009 35483990 PMC9481658

[B148] Sasano Y. Fukumoto K. Tsukamoto Y. Akagi T. Akashi M. (2020). Construction of 3D cardiac tissue with synchronous powerful beating using human cardiomyocytes from human iPS cells prepared by a convenient differentiation method. J. Biosci. Bioeng. 129 (6), 749–755. 10.1016/j.jbiosc.2020.01.001 32151485

[B149] Shao H. Liu A. Ke X. Sun M. He Y. Yang X. (2017). 3D robocasting magnesium-doped wollastonite/TCP bioceramic scaffolds with improved bone regeneration capacity in critical sized calvarial defects. J. Mater Chem. B 5 (16), 2941–2951. 10.1039/c7tb00217c 32263987

[B150] Shen M. Li Y. Lu F. Gou Y. Zhong C. He S. (2023). Bioceramic scaffolds with triply periodic minimal surface architectures guide early-stage bone regeneration. Bioact. Mater 25, 374–386. 10.1016/j.bioactmat.2023.02.012 36865987 PMC9972395

[B151] Shirvan A. R. Nouri A. Wen C. (2021). “Structural polymer biomaterials,” in Structural biomaterials (Elsevier), 395–439.

[B152] Shokouhimehr M. Theus A. S. Kamalakar A. Ning L. Cao C. Tomov M. L. (2021). 3D bioprinted bacteriostatic hyperelastic bone scaffold for damage-specific bone regeneration. Polym. (Basel) 13 (7), 1099. 10.3390/polym13071099 PMC8036866 33808295

[B153] Shuai C. Li Y. Feng P. Guo W. Yang W. Peng S. (2018). Positive feedback effects of Mg on the hydrolysis of poly-l-lactic acid (PLLA): promoted degradation of PLLA scaffolds. Polym. Test. 68, 27–33. 10.1016/j.polymertesting.2018.03.042

[B154] Sikder P. Nagaraju P. Naganaboyina H. P. S. (2022). 3D-Printed piezoelectric porous bioactive scaffolds and clinical ultrasonic stimulation can help in enhanced bone regeneration. Bioengineering 9 (11), 679. 10.3390/bioengineering9110679 36421081 PMC9687159

[B155] Sobral J. M. Caridade S. G. Sousa R. A. Mano J. F. Reis R. L. (2011). Three-dimensional plotted scaffolds with controlled pore size gradients: effect of scaffold geometry on mechanical performance and cell seeding efficiency. Acta Biomater. 7 (3), 1009–1018. 10.1016/j.actbio.2010.11.003 21056125

[B156] Sokolova V. Epple M. (2021). Biological and medical applications of calcium phosphate nanoparticles. Chemistry 27 (27), 7471–7488. 10.1002/chem.202005257 33577710 PMC8251768

[B157] Somo S. I. Akar B. Bayrak E. S. Larson J. C. Appel A. A. Mehdizadeh H. (2015). Pore interconnectivity influences growth factor-mediated vascularization in sphere-templated hydrogels. Tissue Eng. Part C Methods 21 (8), 773–785. 10.1089/ten.tec.2014.0454 25603533 PMC5915224

[B158] Soro N. Attar H. Wu X. Dargusch M. S. (2019). Investigation of the structure and mechanical properties of additively manufactured Ti-6Al-4V biomedical scaffolds designed with a Schwartz primitive unit-cell. Mater Sci. Eng. A 745, 195–202. 10.1016/j.msea.2018.12.104

[B159] Sugawara Y. Kamioka H. Honjo T. Tezuka K. Takano-Yamamoto T. (2005). Three-dimensional reconstruction of chick calvarial osteocytes and their cell processes using confocal microscopy. Bone 36 (5), 877–883. 10.1016/j.bone.2004.10.008 15820146

[B160] Sun H. He S. Wu P. Gao C. Feng P. Xiao T. (2016). A novel MgO-CaO-SiO2 system for fabricating bone scaffolds with improved overall performance. Mater. (Basel) 9 (4), 287. 10.3390/ma9040287 PMC5502980 28773411

[B161] Tamayol A. Bahrami M. (2009). Analytical determination of viscous permeability of fibrous porous media. Int. J. Heat. Mass Transf. 52 (9–10), 2407–2414. 10.1016/j.ijheatmasstransfer.2008.09.032

[B162] Tao J. Zhu S. Liao X. Wang Y. Zhou N. Li Z. (2022). DLP-based bioprinting of void-forming hydrogels for enhanced stem-cell-mediated bone regeneration. Mater today Bio 17, 100487. 10.1016/j.mtbio.2022.100487 PMC9649380 36388461

[B163] Toosi S. Naderi-Meshkin H. Esmailzadeh Z. Behravan G. Ramakrishna S. Behravan J. (2022). Bioactive glass-collagen/poly (glycolic acid) scaffold nanoparticles exhibit improved biological properties and enhance osteogenic lineage differentiation of mesenchymal stem cells. Front. Bioeng. Biotechnol. 10, 963996. 10.3389/fbioe.2022.963996 36159698 PMC9490118

[B164] Toosi S. Naderi-Meshkin H. Kalalinia F. Peivandi M. T. HosseinKhani H. Bahrami A. R. (2016). PGA-incorporated collagen: toward a biodegradable composite scaffold for bone-tissue engineering. J. Biomed. Mater Res. - Part A 104 (8), 2020–2028. 10.1002/jbm.a.35736 27059133

[B165] Torres-Sanchez C. Al Mushref F. R. A. Norrito M. Yendall K. Liu Y. Conway P. P. (2017). The effect of pore size and porosity on mechanical properties and biological response of porous titanium scaffolds. Mater Sci. Eng. C 77, 219–228. 10.1016/j.msec.2017.03.249 28532024

[B166] Truscello S. Kerckhofs G. Bael S. V. Pyka G. Schrooten J. Oosterwyck H. V. (2012). Prediction of permeability of regular scaffolds for skeletal tissue engineering: a combined computational and experimental study. Acta Biomater. 8 (4), 1648–1658. 10.1016/j.actbio.2011.12.021 22210520

[B167] Tsai C.-H. Hung C.-H. Kuo C.-N. Chen C.-Y. Peng Y.-N. Shie M.-Y. (2019). Improved bioactivity of 3D printed porous titanium alloy scaffold with chitosan/magnesium-calcium silicate composite for orthopaedic applications. Mater. (Basel) 12 (2), 203. 10.3390/ma12020203 PMC6356721 30634440

[B168] Valainis D. Dondl P. Foehr P. Burgkart R. Kalkhof S. Duda G. N. Integrated additive design and manufacturing approach for the bioengineering of bone scaffolds for favorable mechanical and biological properties. 2012;1–46. 10.1088/1748-605X/ab38c6 31387088

[B169] Van Bael S. Chai Y. C. Truscello S. Moesen M. Kerckhofs G. Van Oosterwyck H. (2012). The effect of pore geometry on the *in vitro* biological behavior of human periosteum-derived cells seeded on selective laser-melted Ti6Al4V bone scaffolds. Acta Biomater. 8 (7), 2824–2834. 10.1016/j.actbio.2012.04.001 22487930

[B170] Wang G. Chen X. Liu S. Wong C. Chu S. (2016). Mechanical chameleon through dynamic real-time plasmonic tuning. ACS Nano 10 (2), 1788–1794. 10.1021/acsnano.5b07472 26760215

[B171] Wang S. Liu L. Li K. Zhu L. Chen J. Hao Y. Pore functionally graded Ti6Al4V scaffolds for bone tissue engineering application. , 2019;168, 107643, 10.1016/j.matdes.2019.107643

[B172] Wang W. Yeung K. W. K. (2017). Bone grafts and biomaterials substitutes for bone defect repair: a review. Bioact. Mater 2 (4), 224–247. 10.1016/j.bioactmat.2017.05.007 29744432 PMC5935655

[B173] Wang Z. Hui A. Zhao H. Ye X. Zhang C. Wang A. (2020). A novel 3D-bioprinted porous nano attapulgite scaffolds with good performance for bone regeneration. Int. J. Nanomedicine 15, 6945–6960. 10.2147/ijn.s254094 33061361 PMC7520466

[B174] Wei T. Zhang X.-W. Sun H.-Q. Mao M.-Y. (2018). Selective laser sintering and performances of porous titanium implants. J. Stomatol. 36 (5), 532–538. 10.7518/hxkq.2018.05.013 PMC7041146 30465348

[B175] Wildemann B. Kadow-Romacker A. Pruss A. Haas N. P. Schmidmaier G. (2007). Quantification of growth factors in allogenic bone grafts extracted with three different methods. Cell Tissue Bank. 8 (2), 107–114. 10.1007/s10561-006-9021-0 17063261 PMC2795150

[B176] Will J. Ko A. Hopfner U. Aigner J. Wintermantel E. (2004). Ceramic TiO 2 -foams: characterisation of a potential scaffold. J. Eur. Ceram. Soc. 24, 661–668. 10.1016/s0955-2219(03)00255-3

[B177] Williams J. M. Adewunmi A. Schek R. M. Flanagan C. L. Krebsbach P. H. Feinberg S. E. (2005). Bone tissue engineering using polycaprolactone scaffolds fabricated via selective laser sintering. Biomaterials 26 (23), 4817–4827. 10.1016/j.biomaterials.2004.11.057 15763261

[B178] Winarso R. Anggoro P. W. Ismail R. Jamari J. Bayuseno A. P. (2022). Application of fused deposition modeling (FDM) on bone scaffold manufacturing process: a review. Heliyon 8, e11701. 10.1016/j.heliyon.2022.e11701 36444266 PMC9699973

[B179] Wong J. F. Mohan M. D. Young E. W. K. Simmons C. A. (2019). Integrated electrochemical measurement of endothelial permeability in a 3d hydrogel-based microfluidic vascular model. Biosens. Bioelectron. 147, 111757. 10.1016/j.bios.2019.111757 31654819

[B180] Xu M. Zhai D. Chang J. Wu C. (2014). *In vitro* assessment of three-dimensionally plotted nagelschmidtite bioceramic scaffolds with varied macropore morphologies. Acta Biomater. 10 (1), 463–476. 10.1016/j.actbio.2013.09.011 24071000

[B181] Xu Y. Ding W. Chen M. Guo X. Li P. Li M. (2023). Porous iron-reinforced polylactic acid TPMS bio-scaffolds: interlocking reinforcement and synergistic degradation. Mater Des. 231, 112026. 10.1016/j.matdes.2023.112026

[B182] Yan Y. Chen H. Zhang H. Guo C. Yang K. Chen K. (2019). Vascularized 3D printed scaffolds for promoting bone regeneration. Biomater. 190–191, 97–110. 10.1016/j.biomaterials.2018.10.033 30415019

[B183] Ye J. He W. Wei T. Sun C. Zeng S. (2023). Mechanical properties directionality and permeability of fused triply periodic minimal surface porous scaffolds fabricated by selective laser melting. ACS Biomater. Sci. Eng. 9 (8), 5084–5096. 10.1021/acsbiomaterials.3c00214 37489944

[B184] Yenilmez B. Temirel M. Knowlton S. Lepowsky E. Tasoglu S. (2019). Development and characterization of a low-cost 3D bioprinter. Bioprinting 13, e00044. 10.1016/j.bprint.2019.e00044

[B185] Yu Y. Hua S. Yang M. Fu Z. Teng S. Niu K. (2016). Fabrication and characterization of electrospinning/3D printing bone tissue engineering scaffold. RSC Adv. 6 (112), 110557–110565. 10.1039/c6ra17718b

[B186] Yuan L. Ding S. Wen C. (2019). Additive manufacturing technology for porous metal implant applications and triple minimal surface structures: a review. A Rev. 4 (December 2018), 56–70. 10.1016/j.bioactmat.2018.12.003 PMC6305839 30596158

[B187] Yunsheng D. Hui X. Jie W. Tingting Y. Naiqi K. Jiaxing H. (2023). Sustained release silicon from 3D bioprinting scaffold using silk/gelatin inks to promote osteogenesis. Int. J. Biol. Macromol. 234, 123659. 10.1016/j.ijbiomac.2023.123659 36796557

[B188] Zadpoor A. A. (2019a). Mechanical performance of additively manufactured meta-biomaterials. Acta Biomater. 85, 41–59. 10.1016/j.actbio.2018.12.038 30590181

[B189] Zadpoor A. A. (2019b). Additively manufactured porous metallic biomaterials. J. Mater. Chem. B. R. Soc. Chem. 7, 4088–4117. 10.1039/c9tb00420c 31701985

[B190] Zerankeshi M. M. Bakhshi R. Alizadeh R. (2022). Polymer/metal composite 3D porous bone tissue engineering scaffolds fabricated by additive manufacturing techniques: a review. Bioprinting 25, e00191. 10.1016/j.bprint.2022.e00191

[B191] Zhang J. Wehrle E. Adamek P. Paul G. R. Qin X.-H. Rubert M. (2020). Optimization of mechanical stiffness and cell density of 3D bioprinted cell-laden scaffolds improves extracellular matrix mineralization and cellular organization for bone tissue engineering. Acta Biomater. 114, 307–322. 10.1016/j.actbio.2020.07.016 32673752

[B192] Zhang L. Feih S. Daynes S. Chang S. Wang M. Y. Wei J. (2018). Energy absorption characteristics of metallic triply periodic minimal surface sheet structures under compressive loading. Addit. Manuf. 23, 505–515. 10.1016/j.addma.2018.08.007

[B193] Zhang Q. Zhou J. Zhi P. Liu L. Liu C. Fang A. (2023). 3D printing method for bone tissue engineering scaffold. Med. Nov. Technol. Devices 17, 100205. 10.1016/j.medntd.2022.100205 PMC9995276 36909661

[B194] Zhang Y. Zhu Y. Chen F. (2016). Novel interconnected nanochannel hydroxyapatite ceramics: synthesis, microstructure, and permeability. Ceram. Int. 43, 5403–5411. 10.1016/j.ceramint.2016.12.113

[B195] Zhianmanesh M. Varmazyar M. Montazerian H. (2019). Fluid permeability of graded porosity scaffolds architectured with minimal surfaces. ACS Biomater. Sci. Eng. 5 (3), 1228–1237. 10.1021/acsbiomaterials.8b01400 33405642

[B196] Zhou X. Zhou G. Junka R. Chang N. Anwar A. Wang H. (2021). Fabrication of polylactic acid (PLA)-based porous scaffold through the combination of traditional bio-fabrication and 3D printing technology for bone regeneration. Colloids Surfaces B Biointerfaces 197, 111420. 10.1016/j.colsurfb.2020.111420 33113493 PMC7738389

[B197] Zhu J. Zou S. Mu Y. Wang J. Jin Y. (2022). Additively manufactured scaffolds with optimized thickness based on triply periodic minimal surface. Mater. (Basel) 15 (20), 7084. 10.3390/ma15207084 PMC9605549 36295151

[B198] Zhu L.-Y. Li L. Shi J.-P. Li Z.-A. Yang J.-Q. (2018). Mechanical characterization of 3D printed multi-morphology porous Ti6Al4V scaffolds based on triply periodic minimal surface architectures. Am. J. Transl. Res. 10 (11), 3443–3454. 30662598 PMC6291701

